# Microbiome diversity and metabolic capacity determines the trophic ecology of the holobiont in Caribbean sponges

**DOI:** 10.1038/s43705-022-00196-3

**Published:** 2022-11-10

**Authors:** Michael P. Lesser, M. Sabrina Pankey, Marc Slattery, Keir J. Macartney, Deborah J. Gochfeld

**Affiliations:** 1 Department of Molecular, Cellular and Biomedical Sciences, University of New Hampshire, Durham, NH 03824 USA; 2 Department of BioMolecular Sciences, Division of Pharmacognosy, University of Mississippi, Oxford, MS 38677 USA; 3 National Center for Natural Products Research, University of Mississippi, Oxford, MS 38677 USA; 4 Present Address: University of Texas Rio Grande Valley, School of Earth, Environmental and Marine Sciences, Port Isabel, TX 78958 USA

**Keywords:** Microbial ecology, Stable isotope analysis

## Abstract

Sponges are increasingly recognized as an ecologically important taxon on coral reefs, representing significant biomass and biodiversity where sponges have replaced scleractinian corals. Most sponge species can be divided into two symbiotic states based on symbiont community structure and abundance (i.e., the microbiome), and are characterized as high microbial abundance (HMA) or low microbial abundance (LMA) sponges. Across the Caribbean, sponge species of the HMA or LMA symbiotic states differ in metabolic capacity, as well as their trophic ecology. A metagenetic analysis of symbiont 16 S rRNA and metagenomes showed that HMA sponge microbiomes are more functionally diverse than LMA microbiomes, offer greater metabolic functional capacity and redundancy, and encode for the biosynthesis of secondary metabolites. Stable isotope analyses showed that HMA and LMA sponges primarily consume dissolved organic matter (DOM) derived from external autotrophic sources, or live particulate organic matter (POM) in the form of bacterioplankton, respectively, resulting in a low degree of resource competition between these symbiont states. As many coral reefs have undergone phase shifts from coral- to macroalgal-dominated reefs, the role of DOM, and the potential for future declines in POM due to decreased picoplankton productivity, may result in an increased abundance of chemically defended HMA sponges on tropical coral reefs.

## Introduction

Recent declines of coral cover, due to a variety of natural and anthropogenic stressors [1, 2], has resulted in a significant loss of biodiversity, ecosystem function, and important ecosystem services for coral reefs around the world [2, 3]. Shallow (<30 m) coral reefs have undergone phase shifts due to climate change-related coral bleaching and disease, as well as overfishing and coastal degradation, resulting in communities that are increasingly dominated by alternative competitors such as algae, anemones, corallimorphs, octocorals, and sponges [4].

Sponges are seen as an emerging, and dominant, taxon on many coral reefs [5, 6], both in terms of biomass and biodiversity, and these changes have the potential to alter the functional diversity of coral reefs [7]. These observations resulted in the hypothesis that sponges would become increasingly more dominant as coral abundance and biodiversity decline [5]. It has also been argued, however, that climate change-related modifications in the physical oceanography of tropical coral reefs could lead to decreases in net primary productivity, specifically of picoplankton [8]. This could lead to food limitation for an increasing population of filter-feeding sponges, potentially limiting their predicted increase on coral reefs [8]. To appreciate the potential for sponges to become competitively dominant on coral reefs, we should understand and quantify the capabilities of sponges to exploit newly available habitat. One trait that enables sponges to exploit new habitats is their symbiotic state, an emergent property of sponge host-microbial co-evolutionary histories that determines the functional ecology of sponges [9] and affects the ecological outcomes for sponges on coral reefs [10].

The Caribbean basin has a very diverse sponge fauna that contains many species of heterotrophic and photoautotrophic sponges [11], whose functional ecology is essential for the health of coral reef communities [12]. The significant role that sponges play in benthic-pelagic coupling on coral reefs [13–15], and the chemical ecology of sponges that results in the biosynthesis of secondary metabolites for defense against predators, competitors, and pathogens [16, 17], both contribute to the success of sponges on coral reefs.

Many coral reef sponges host complex assemblages of symbiotic microbes with diverse metabolic capabilities (i.e., microbiome), dominated by Bacteria and Archaea that are distinct from the microorganisms in the surrounding seawater [18, 19]. Some sponges are described as high microbial abundance (HMA) sponges, supporting large and diverse microbial communities [19–22] that have critical roles in macronutrient (e.g., nitrogen, phosphorus, sulphur) cycling on coral reefs [23–25], as well as the production of defensive secondary metabolites [16, 17]. In contrast, the microbial communities in low microbial abundance (LMA) sponges are less diverse [22, 26], but still provide important functions for the sponge holobiont [18, 25]. Differences in microbial abundance have implications for sponge trophic ecology. Specifically, HMA sponges have increased mesohyl density, low choanocyte densities, and reduced mass-specific pumping rates, whereas LMA sponges have decreased mesohyly density, higher choanocyte densities and greater mass-specific pumping rates [27]. These differences are also associated with preferential uptake of dissolved organic matter (DOM) in HMA sponges and particulate organic matter (POM) in LMA sponges [27]. Recent work has demonstrated that coral reef sponges can play a significant role in the transformation of DOM into a detrital pathway via the “sponge loop” [28, 29], which describes sponges as important sinks for DOM on coral reefs. The sponge loop hypothesis postulates that sponges consume large amounts of DOM, which includes both dissolved organic carbon (DOC) and dissolved organic nitrogen (DON) that is used as a food resource and then released as detritus in the form of choanocytes that feed higher trophic levels and could have significant impacts on carbon fluxes and budgets on coral reefs [29].

Given that significant amounts of fixed carbon from corals and benthic algae can be released into seawater as DOM, and 50% of all DOM is derived from the exudate or lysis of primary producers, there is a considerable pool of DOM to be exploited. With a molar C:N ratio >10 in most cases [30, 31], sponges are never carbon limited, and while DOM provides both energy and carbon skeletons for protein synthesis, the latter process still requires the intake of nitrogen, making the consumption and assimilation of DON and particulate organic nitrogen (PON) essential for sponge growth [10]. Compared to DOM, however, POM is significantly more bioavailable [32], and sponges efficiently consume PON primarily from the bacterioplankton portion of POM with clearance rates of 83–90% [10, 13, 33]. These bacterioplankton have low molar C:N ratios of ~4–6 and are a well-known source of nitrogen for active suspension feeders [34–36]. While bioavailable POM can supply nitrogen directly to the host, it is likely that the microbiome obtains most of its nitrogen requirements in the form of dissolved inorganic nitrogen (DIN) from the nitrogen waste products of the host consumption and metabolism of POM and DOM [36].

Sponges, and their microbiomes, have well-known roles in nitrogen and carbon cycling on coral reefs [23, 29, 36], and it is increasingly clear that the ecological success of sponges is a function of both the host and its microbiome, as well as their interactions. The emergent properties of sponges and their microbiomes also have significant implications for the functional ecology and biodiversity of coral reef ecosystems. The primary question addressed here is whether symbiotic state determines the trophic ecology of sponge holobionts. By evaluating the differences in microbiome communities of several Caribbean sponges representing both HMA and LMA symbiotic states, and their functional capacities and trophic ecology using metagenomics and stable isotopic analyses, important insights into the ecological roles of HMA versus LMA sponges on coral reefs can be inferred.

## Materials and methods

### Study sites and sponge collections

To evaluate the phylogenetic, genetic, and functional diversity of sponges across the broader Caribbean basin, replicate sponges were collected at the same depth (15 m) from 3–4 reef sites within each of the following locations: Belize, Curaçao, Grand Cayman and St. Croix USVI (Table S1). The sponges represent both HMA and LMA states, as well as with and without photosymbionts or chemical defenses. Samples of individual sponges were collected into individual resealable plastic bags at each of these locations (*n* = 5 replicates for each species/location/reef site combination: ~300 samples), subsampled into cryovials and preserved in a DNA preservation buffer [37] and samples for RNA extraction preserved in RNALater®, and frozen (−20 °C to −80 °C initial freezing temperature) for transport to the University of New Hampshire for analysis. The targeted sponge species were sourced from different sponge functional groups representing different relative bacterial abundances, the presence or absence of photoautotrophic symbionts, and differential production of chemical defenses (Table 1). Specifically, we examined *Aplysina cauliformis*, *Amphimedon compressa*, *Niphates erecta*, *Xestospongia muta* and *Agelas conifera/tubulata*. The latter species represents two phenotypic morphotypes that are genetically indistinguishable [9] and were analyzed together in this study. These genera are widely distributed throughout the Caribbean and therefore the results from these taxa will be broadly generalizable to their respective functional groups. Specific reef sites were chosen to be as ecologically similar as possible across all locations and abiotic characteristics (e.g., temperature) at the time of sponge collection have been reported previously [38].

**Table 1:** Selected sponge genera for field collections in the Caribbean based on their bacterial phenotype [21]^1^, presence of photosymbionts [9]^2^, and evidence for being chemically defended [16]^3^.

Sponge species	^1^Bacterial phenotype	^2^Photosymbionts	^3^Chemically defended
*Aplysina cauliformis*	HMA	yes	yes
*Agelas conifera/tubulata*	HMA	no*	yes
*Xestospongia muta*	HMA	yes	variable
*Amphimedon compressa*	LMA	yes	yes
*Niphates erecta*	LMA	yes	no

*Agelas spp. have been found to contain very low numbers of cyanobacterial symbionts based on amplified sequence variants of 16 S rRNA [9] unlikely to be of functional significance.

### DNA extraction

DNA for both the 16 S rRNA and metagenome libraries was extracted from sponge tissue samples (~200 mg) using the DNEasy PowerSoil® DNA isolation kit (Qiagen; Hilden, Germany), following the manufacturer’s instructions with modifications for cell lysis as described by Sunugawa et al. [39]. This protocol was used for all DNA extractions as follows: incubation in 5 µl 10 mg ml^−1^ Proteinase K, 0.19 µl 10 U µl^−1^ Lysozyme and 2 µl RNAse A at 55 °C for 12 h, followed by two rounds of 2 min bead-beating using a Qiagen QuickLyser set at 50 Megahertz. Purified gDNA was assessed for quality and concentration using a NanoDrop 2000c spectrophotometer.

### 16 S rRNA metagenetic libraries

Microbial DNA was amplified using the polymerase chain reaction (PCR) with primer sets targeting the universal bacterial/archaeal 16 S rRNA gene. Samples were amplified with new degenerate primers designed to amplify the 16 S rRNA gene (hypervariable region V3-V4), consisting of the forward primer 515 F (5′-GTG YCA GCM GCC GCG GTA A-3′; [40]) and the reverse primer 806 R (5′-GGA CTA CHV GGG TWT CTA AT-3′; [41]). Fluidigm linker sequences CS1 (5′-ACA CTG ACG ACA TGG TTC TAC A-3′) and CS2 (5′-TAC GGT AGC AGA GAC TTG GTC T-3′) were added to the 5’ end of both forward and reverse primers to facilitate Illumina MiniSeq. The 16 S rRNA gene PCR consisted of a 25 μl reaction with 12.5 μl AmpliTaq Gold 360 Master Mix (Applied Biosystems), 1.0 μl GC-enhancer, 0.5 μl 515 F (10 μM) and 0.5 μl 806 R (10 μM), 2.0 μl of DNA template (40–60 ng), and 8.5 μl nuclease free water (Integrated DNA Technologies, Coralville, Iowa). Triplicate reactions were performed for each sponge sample and pooled using the following protocol: initial denaturation for 10 min at 95 °C, 30 cycles at 95 °C for 30 s, 50 °C for 60 s, and 72 °C for 60 s, followed by a 10 min extension at 72 °C. The PCR products were then electrophoresed on a 1% agarose gel. The 16 S rRNA PCR amplicons containing Fluidigm linkers were sequenced on an Illumina MiniSeq System employing V2 chemistry (2 × 150 bp reads, mean 45388 read-pairs per sample) at the University of Illinois at Chicago (UIC) Research Resources Center’s Sequencing Core. Amplicon sequence variants (ASVs) were identified and tabulated across samples using DADA2 v1.14 [42]. Briefly, raw reads were trimmed from the initial 20 bp to remove residual primer, and then truncated beyond the first instance of quality scores below 3 (truncQ = 2). The maximum expected error during denoising (maxEE) was 2 and 5 for forward and reverse reads, respectively. The error model was built from the first 100 M bases and inspected using ‘plotErrors’. Denoised reads were then merged and chimeric contigs discarded using mergePairs and removeBimeraDenovo, respectively. Taxonomic ranks were assigned to the inferred ASVs using the SILVA ribosomal reference database release 132, and the DADA2 function ‘assignTaxonomy’.

### Microbiome composition analyses

Analysis of the sponge and environmental microbial communities was facilitated by *phyloseq* functions in R [43]. The ASV count table was first filtered to discard samples with fewer than 8000 counts and then filtered to retain ASVs detected in more than one sample and accounting for at least 10 occurrences across samples. ASVs assigned to the Order “Chloroplast” by SILVA taxonomy were excluded from further analysis. Sample counts were then rarefied to normalize for sequencing effort.

#### Alpha and beta diversity of sponge microbiomes

Shannon diversity of 16 S rRNA communities among samples was quantified using the ‘diversity’ function from vegan [44]. Effects of host species and location on overall Shannon diversity were assessed using two-way ANOVA on normalized ASV counts using the trimmed mean of M-values (TMM) method in edgeR. Subsequently, effect of symbiotic state (HMA, LMA) was tested using a linear mixed-effects (LME) model to control for species, with symbiotic state as fixed effect and species as random effect, using the function ‘lme’ from the R package nmle [45]. Within sampling location effect was also tested with an LME model, with location as a random effect. Effects of location and species on 16 S rRNA composition were evaluated using single-factor PERMANOVAs with the ‘adonis2’ function from the vegan package, with Bray-Curtis distances among samples calculated from normalized counts. Pairwise contrasts between species and locations were examined using ANOSIM. Composition was assessed at two levels: ASV counts (4274 ASVs) and counts agglomerated to microbial class (60 classes). Effect of symbiotic state was tested using a nested PERMANOVA to control for species with the function ‘np.manova’ from the R package BiodiversityR [46]. The differential enrichment of 16 S rRNA ASVs between sponge species, localities and symbiotic states was assessed using Wald tests through the R package DESeq2 [47].

### Metagenome library preparation

Metagenome libraries were constructed from 57 samples, consisting of *Agelas conifera/tubulata* (HMA, *n* = 3)*, Amphimedon compressa* (LMA, *n* = 3)*, Aplysina cauliformis* (HMA, *n* = 3)*, Niphates erecta* (LMA, *n* = 3), and *Xestospongia muta* (HMA, *n* = 3) collected from all four locations, except for *A. compressa*, which was only collected from Belize, Grand Cayman, and St. Croix. Libraries were constructed using the protocol for NEBNext Ultra II FS DNA library preparation (New England Biolabs) and sequenced on the Illumina HiSeq2500 platform (PE150; Novogene). Reads from demultiplexed sequence files were error-trimmed using Trimmomatic [48] and mapped to the UniRef database using PALADIN [49]. Enzyme-mapped read-counts to enzymes using the 175 Kyoto Encyclopedia of Genes and Genomes (KEGG) metabolic pathways were then tabulated according to either metazoan, bacterial, or archaeal origin using PALADIN plugins *‘taxonomy*’ and *‘pathways’*. Enzyme-mapped read counts were then normalized to account for sample variance using the trimmed mean of M-values (TMM) method available from the R package edgeR [50]. Metagenomic libraries are available at the NCBI Short Read Archive under BioProject PRJNA555077. To quantify genomic clusters associated with secondary metabolite production, metagenome samples were pooled by species for assembly using MegaHit v1.1.3 [51]. Sample reads were mapped to each species assembly using BWA v0.7.17-r1188 [52]. Secondary metabolite clusters were then identified and tabulated using AntiSMASH v4.1.0 [53].

### Alpha and beta diversity of metagenomes

Shannon diversity indices for the contribution to functional capacity in the metabolic pathways of sponges were calculated using the ‘diversity’ function from vegan [44]. Effects of host species and location on overall Shannon diversity were assessed using two-way ANOVA on normalized enzyme-mapped read counts. Subsequently, counts mapping to Archaea, Bacteria, and Metazoa were isolated and tested for domain-specific patterns. Effects of site and species on the contribution of different metabolic pathways were evaluated using single-factor PERMANOVAs with the ‘adonis2’ function from the vegan package, with Bray-Curtis distances among samples calculated from normalized enzyme-mapped read counts. Compositional differences were assessed first across the 4853 KEGG enzymes using ANOSIM initially, and then separately for enzyme-mapped read counts within each pathway using PERMANOVAs, followed by false discovery rate (FDR) correction of *p*-values.

### Effect of symbiotic state on sponge functional capacity

Differential enrichment in the functional capacity of several metabolic pathways between HMA and LMA sponge samples was assessed using Wald tests, on the cumulative counts of enzyme-mapped read counts from each pathway, through the R package DESeq2 [47]. The metagenomic analysis of specific groups of functional genes involved in the cycling of carbon and nitrogen were used to assess heterotrophic versus photoautotrophic capacity, and genes involved in secondary metabolite biosynthetic pathways (*e.g*., polyketide synthases) were assessed to determine the capacity for chemical defense production in sponges.

### Stable isotope analyses

The abundance of natural stable isotopes (C and N) in sponge tissue for each species, from each location, were used as indicators of sponge dependence on the uptake of autotrophically sourced exogenous DOM. This DOM comes from autotrophic sources including corals, macrophytes and lysed phytoplankton versus POM, primarily sourced from picoplankton including autotrophic and heterotrophic bacteria. Subsamples of the sponge holobionts were frozen in aluminum foil for transport, freeze-dried and washed so that only organic material was analyzed. Sponge samples were placed onto pre-combusted (450 °C, 6 h) GF/F filters (0.7 µm) and combusted in a Carlo Erba NA 1500 elemental analyzer interfaced with a Delta Plus mass spectrometer and analyzed for particulate C and N, as well as for δ^15^N and δ^13^C stable isotopes. Pre-combusted GF/F filters were also processed as blanks to account for any background signal. In addition to parametric statistics, Stable Isotope Bayesian Ellipses in R (SIBER) [54, 55], was used to determine the size and overlap of sponge trophic niches for individual sponges and competition for food resources using bulk stable isotope values for both δ^13^C and δ^15^N per mil (‰).

## Results

### Alpha and beta diversity of microbiomes

The Shannon H index of microbial alpha, or species, diversity differed among species and sampling sites (ANOVA, site F_3,323_ = 10.0, *p* < 0.001; species F_4,323_ = 646.8, *p* < 0.001). All species (Table 1) differed significantly in *post hoc* pairwise Tukey’s HSD comparisons, except *Aplysina cauliformis* and *Xestospongia muta*, both HMA species with photosymbionts (i.e., cyanobacteria). All HMA species exhibited consistently higher microbiome diversity than LMA species, and within the latter, *Niphates erecta* produced the lowest Shannon index. Overall, HMA alpha diversity was significantly higher than LMA diversity when accounting for individual species effects (nested ANOVA F_1,3_ = 19.1, *p* = 0.02), with no significant effect of location (nested ANOVA F_13,314_ = 1.1, *p* = 0.35) observed (Fig. S1).

**Fig. 1:**
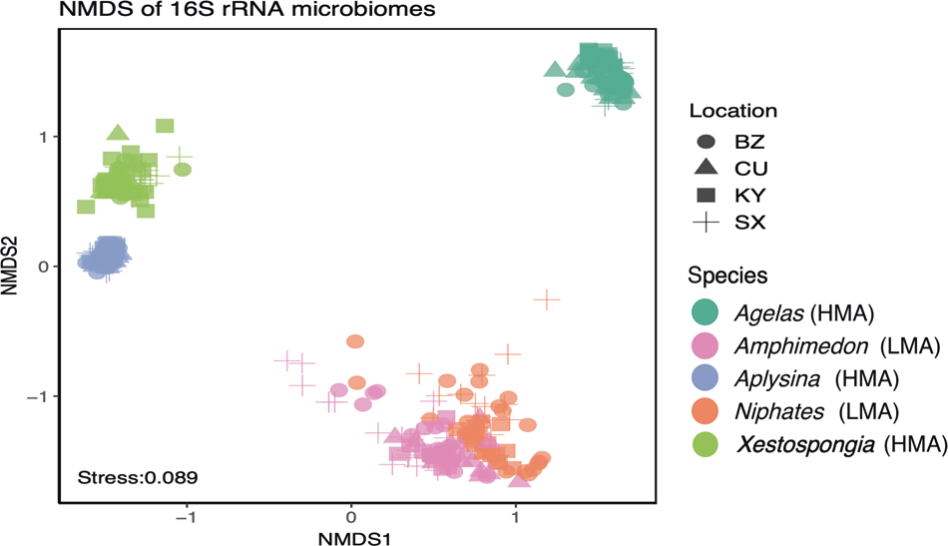
Beta diversity and ordination of microbial community samples. Samples colored by species and microbiome symbiotic states; High Microbial Abundance (HMA) and Low Microbial Abundance (LMA), with shape denoting sample location (BZ = Belize, CU = Curaçao, KY = Grand Cayman, SX = St. Croix).

The beta diversity for microbial community composition at the amplicon sequence variants (ASV) (4210 ASVs) and class levels (60 classes) were significantly affected by both sponge species (PERMANOVA F_4,326_ = 189.5, *p* = 0.001 for ASV; F_4,326_ = 402, *p* = 0.001 for class) and sampling location (PERMANOVA F_3,327_ = 3.22, *p* = 0.001 for ASV; F_3,327_ = 2.4, *p* = 0.031 for class) (Fig. 1), where species explains 70% and 83% of the variance for ASV and class, respectively, but location explains significantly less (<3%) of the variance for ASV and class. When controlling for sponge species variance, there was not a significant difference (nested PERMANOVA F_1,326_ = 2.1, *p* = 0.14) based on microbiome composition despite the observed segregation of HMA and LMA symbiotic states (Fig. 1). Highly significant differences in microbiome diversity were detected using ANOSIM pair-wise comparisons between all species (ANOSIM R ≥ 0.95, *p* = 0.001), while location differences produced lower dissimilarities (R < 0.04). Overall, microbial composition in sponges from Belize, St Croix and Curaçao all differed from each other, while those from Grand Cayman did not differ significantly from any other locality.

### Microbiome differences between symbiotic states

A total of 587 ASVs were significantly enriched in HMA sponges (notably: *Chloroflexi* [photoheterotrophic], *Gemmatimonadetes* [photoheterotrophic], *Acidobacteria* [heterotrophic/carbon utilization], *Poribacteria* [mixotrophic/carbon fixation]), and 417 ASVs were enriched in LMA sponges (including: *Bacteriodetes* [protein and complex carbohydrate degradation], *Planctomycetes* [anaerobic ammonium oxidation: ANAMMOX] and *Cyanobacteria* [photoautotrophic]) (Fig. S2). Locations (*n* = 4) were differentially enriched for few ASVs, with no location enriched for more than 88 ASVs in pairwise contrasts. Most enriched variants belonged to *Proteobacteria* and *Chloroflexi*.

**Fig. 2:**
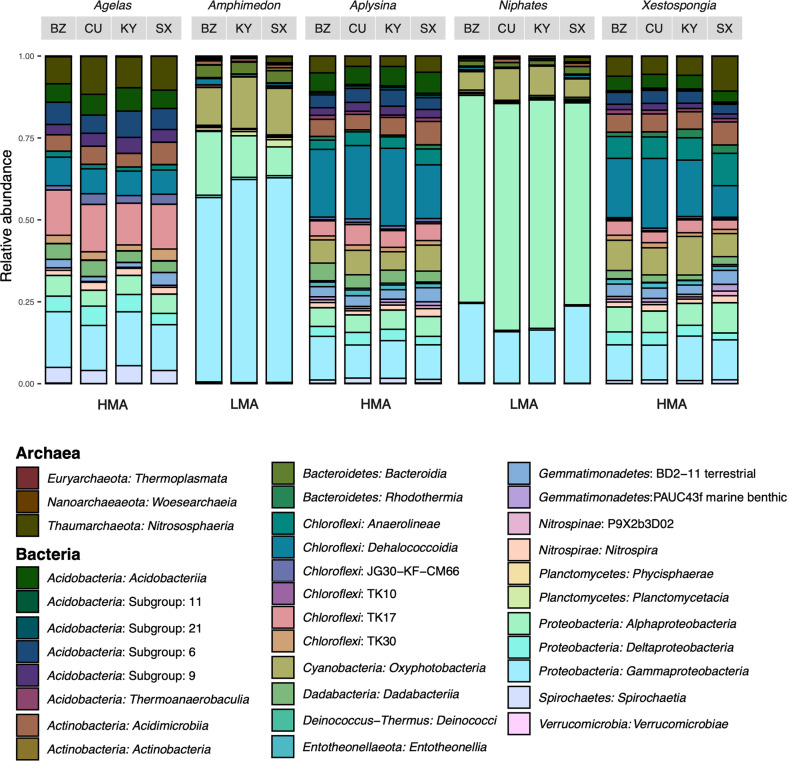
Relative abundances of microbial classes, averaged within species at each collection location (BZ = Belize, CU = Curaçao, KY = Grand Cayman, SX = St. Croix) and microbome symbiotic states; High Microbial Abundance (HMA) or Low Microbial Abundance (LMA). Only classes accounting for at least 1% of reads are shown. HMA high microbial abundance, LMA low microbial abundance.

Host species (*n* = 5) enrichments followed the trends observed for HMA/LMA enrichment patterns (Fig. 2). For comparisons within host symbiotic state (i.e., HMA versus LMA), there were fewer overall ASVs differing between species. *Xestospongia muta* and *Aplysina cauliformis* were both enriched for ASVs belonging to *Chloroflexi*, *Actinobacteria* (saprophytic), *Spirochaetes* (chemoheterotrophic/nitrogen fixation), *Acidobacteria* and *Gemmatimonadetes*, when compared to *Agelas conifera/tubulata*. The LMA species, *Niphates erecta* and *Amphimedon compressa*, were differentially enriched by only 101 and 184 ASVs respectively, with *Proteobacteria* representing the majority of differential enriched ASVs.

### Metabolic diversity from metagenomes

**Fig. 3:**
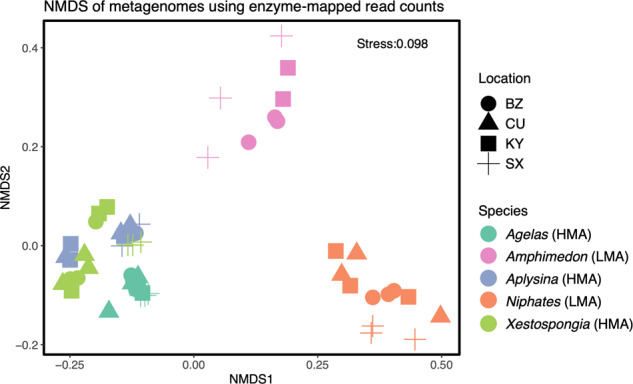
Ordination of metagenomes for all species and locations of collection based on Bray-Curtis distances of total counts detected for each KEGG pathway. Samples colored by species and microbiome symbiotic states; High Microbial Abundance (HMA) and Low Microbial Abundance (LMA), with shape denoting sample location (BZ = Belize, CU = Curaçao, KY = Grand Cayman, SX = St. Croix).

The metagenome library size was 29,771,675 read-pairs ± 4,798,947 (mean ± SD). When these reads were queried against genes encoding 175 KEGG pathways, 157 were detected and a total of 4853 enzymes identified. Host species had a significant effect on overall metabolic diversity, as did Domain, with Bacteria consistently showing greater metabolic diversity regardless of site (Fig. S3). Based on Shannon’s H diversity index, host species predicted that metabolic differences were driven by sponge symbiotic state, with HMA sponges showing significant differences from, and greater functional metabolic capacity than, LMA sponges (ANOVA F_1,53_ = 13.3, *p* < 0.001) (Fig. 3). Tukey’s HSD *post hoc* multiple comparisons revealed that this effect was largely driven by LMA species with less-diverse microbiomes (*A. compressa* and *N. erecta*) which differed from each other and from all HMA species. Functional differences in metabolic capacity, at both the metabolic process and pathway levels, were driven primarily by species differences (PERMANOVA F_4,50_ = 12.6, *p* = 0.001), with all HMA species grouping together and LMA species showing significant differences compared to HMA species (Fig. 3). While location was not a significant factor overall, tests on individual pathways revealed 125 pathways where enzyme composition was affected by species differences, and only 14 pathways were affected by location (Table S2).

### Metabolic pathway differences between HMA and LMA sponges

The composition of KEGG metabolic enzymes detected from metagenomic reads differed between HMA and LMA sponge samples (ANOSIM R = 0.81 *p* = 0.001) (Fig. 3). HMA sponges exhibited greater metabolic alpha diversity (Shannon H) across the 4853 enzymes (ANOVA F_1,53_ = 267, *p* < 0.001) (Fig. S3). Symbiotic states (HMA/LMA) differed in enzymatic composition for 121/157 KEGG pathways (FDR-adjusted PERMANOVAs, Table S2). Sampling location did not affect composition for any pathways after FDR correction. The relative contribution of microbes (Archaea and Bacteria) to an HMA host’s functional capacity was significantly higher than for LMA sponges across all metabolic pathways (Wilcoxon sign-rank V = 9515, *p* < 0.0001). For the metabolic pathways examined, the enzymes in those pathways came from a broader diversity of microbial taxa in HMA sponges, mirroring the broader microbial diversity observed within HMA sponges (Fig. S4). For example, Archaea contributed reads to amino acid (valine/leucine) and energy (photosynthesis) metabolism as well as to streptomycin biosynthesis in HMA sponges, and to ansamycin biosynthesis in LMA sponges. However, Bacteria provided increased functional redundancy across most metabolic categories in HMA but not LMA sponges (Fig. S4).

**Fig. 4:**
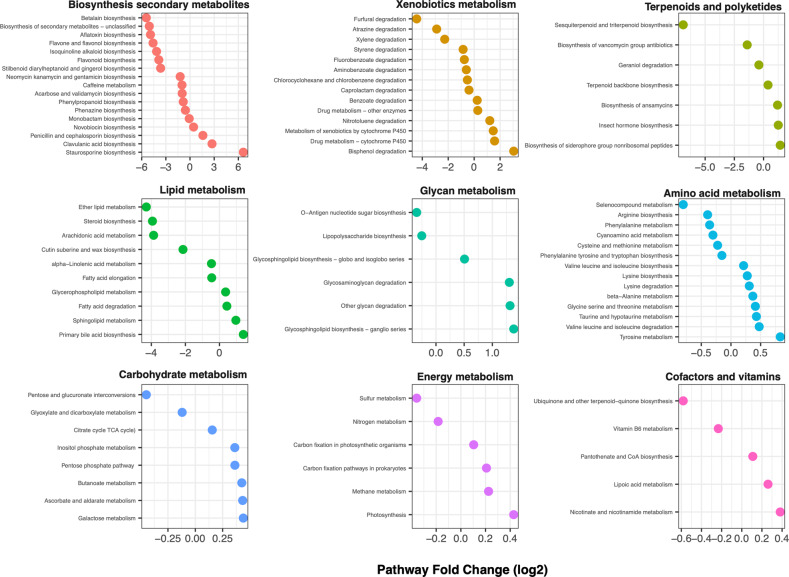
Differential enrichment of metabolic pathways detected between high microbial abundance (HMA) and low microbial abundance (LMA) microbiome symbiotic states. logFC < 0: HMA-enriched, logFC > 0: LMA-enriched. Only pathways with significant differences under Wald tests (adjusted *p* < 0.05) are shown.

**Fig. 5:**
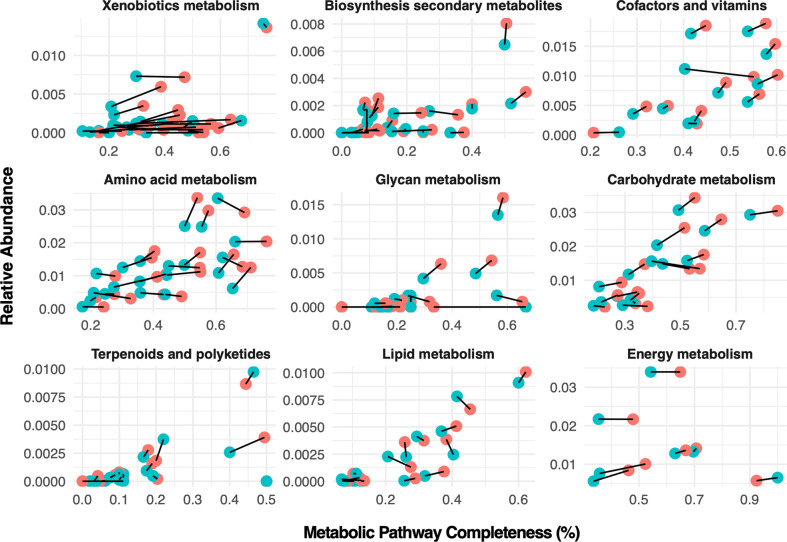
Relative contribution of microbial and host genomes to total pathway abundances. Relative contributions are averaged for High Microbial Abundance (HMA) states in red, and Low Microbial Abundance (LMA) symbiotic states in blue.

When 157 KEGG pathways are examined based on HMA-LMA states, there were 87 pathways with differential enrichment in terms of relative abundance and/or enzymatic completeness for a metabolic pathway. In terms of enzyme abundances based on enzyme-mapped read counts, 43 pathways were significantly enriched in HMA and 44 enriched in LMA sponges based on Wald tests, although most did not differ significantly in magnitude (e.g., log2 fold-change values <1) (Fig. 4, Table S2). HMA sponges were especially enriched for pathways involving secondary metabolite production (Wald test adjusted-*p* < 0.05: 13 HMA-enriched vs 4 LMA-enriched pathways), while carbohydrate metabolism pathways were more likely to be enriched in LMA pathways (6 significantly LMA-enriched vs 2 HMA-enriched). In terms of pathway completeness (i.e., percentage of enzymes detected per KEGG path), HMA sponges exhibited higher average pathway completeness than LMA sponges (45.4% vs 32.3%: Welch’s *t* = 9.8, df = 42, *p* < 0.0001), notably among metabolic pathways involving amino acids, xenobiotics, secondary metabolites, vitamin biosynthesis, carbohydrate metabolism and energy production (carbon, sulfur, methane, nitrogen cycling) (Figs. 5,  S5).

While the enzyme-mapped read counts involved in prokaryotic carbon fixation (KEGG EC00720) were ~15% more abundant in LMA sponges (Fig. 4, Table S2), the reads from HMA sponges accounted for more component enzymes (33 enzymes detected in HMA samples compared to 27 detected in LMA samples, on average) (ANOVA F_1,53_ = 64, *p* < 0.001) (Figs. 5,  S5). Specifically, HMA sponges were enriched for enzymes involved in the reductive citrate cycle, the 3-hydroxypropionate cycle and the hydroxyproprionate-hydroxybutyrate cycles, while LMA sponges were enriched for enzymes in the Wood-Ljungdahl cycle (i.e., the reductive acetyl CoA pathway; Fig. S6). Enzymes involved in oxygenic photosynthesis were detected in both HMA and LMA samples, both at relatively low abundances (<1% of mapped reads) compared with abundances of reads mapped to other prokaryotic carbon fixation pathways described above (3.5% of all mapped reads).

HMA sponges were also enriched for multiple KEGG pathways involving the biosynthesis of secondary metabolites including sesquiterpenes, triterpenes, betalain, aflatoxin, flavonoids, isoquinoline alkaloids, as well as steroids, vitamins, and degradation of xenobiotics. Among glycan (i.e., polysaccharide) metabolic processes, HMA samples contained more enzymes involved in lipopolysaccharide biosynthesis, while LMA samples were enriched for glycosaminoglycan (GAG) metabolic pathways. Significantly more genomic clusters were also identified in HMA sponges for 24/30 secondary metabolite categories compared with LMA sponges using AntiSMASH (Welch’s *t*-tests, Bonferroni-adjusted *p*-values < 0.05) (Fig. S7) Notably, HMA sponges were highly enriched for gene clusters involved in terpene biosynthesis, type I polyketide synthases and bacteriocins. LMA sponges were more enriched for KEGG secondary metabolite pathways involving staurosporine, clavulanic acid, and siderophore-group non-ribosomal peptides (Table S2), but were not enriched for any of the broader metabolite categories using AntiSMASH (Fig. S7).

### Stable isotope differences between sponges by species and symbiotic state

The natural abundance of δ^13^C ‰ and δ^15^N ‰ in the tissue (i.e., holobiont) of sponges across the Caribbean (Fig. S8) show a significant effect of species for both δ^13^C (ANOVA: F_4,161_ = 84.12, *p* < 0.0001) and δ^15^N (ANOVA: F_4,161_ = 7.08, *p* < 0.0001). The two known photoautotrophic sponges, *Aplysina cauliformis* and *Xestospongia muta*, had the lowest δ^13^C values, and were significantly different from each other, and from all other sponge species (Fig. S8, Tukey’s HSD, *p* < 0.05). For the δ^15^N values there was significantly more overlap between species with *A. cauliformis* significantly different (Tukey’s HSD, *p* < 0.05) from all other species (Fig. S8). The C:N ratios for sponge species were also significant (ANOVA: F_4,161_ = 86.41, *p* < 0.0001) with all values indicating nutrient sufficiency. Multiple comparison testing did reveal that *X. muta* had the highest C:N ratio at 5.34, which was significantly different than the lowest C:N ratio seen in *A. tubulata/conifera* at 3.89. When these same sponge species were compared based on their symbiotic state a significant difference (ANOVA: F_1,161_ = 70.26, *p* < 0.0001) for δ^13^C ‰ was observed with HMA sponges having more negative (−18. 7 ± 0.08 [SE]) δ^13^C ‰ values than LMA sponges (−17.6 ± 0.09 [SE]) with a Cohen’s d effects size test of 1.35. For the δ^15^N values there were no significant (ANOVA: F_1,161_ = 0.83, *p* = 0.774) differences between HMA (7.43 ± 0.29 [SE]) and LMA species (6.24 ± 0.16 [SE]) with a Cohen’s d effects size test of 0.06. The C:N ratios of HMA sponges (4.59 ± 0.06) were significantly (ANOVA: F_1,161_ = 4.58, *p* = 0.034) greater than LMA sponges (4.41 ± 0.04) but with a weak Cohen’s d effects size of 0.23 and a non-significant adjusted *p*-value of 0.051.

**Fig. 6:**
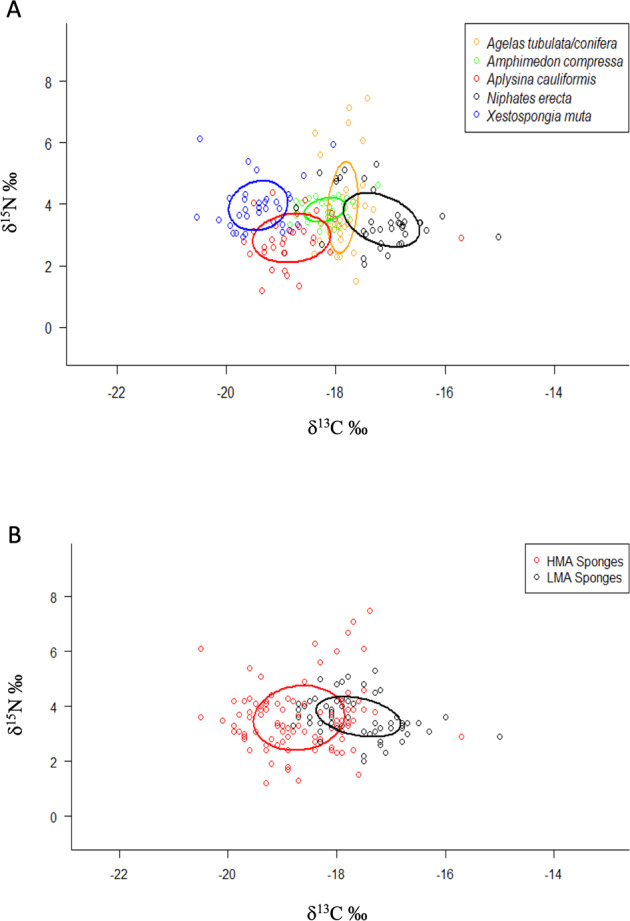
Bivariate plot of SIBER analysis, using the δ^13^C and δ^15^N from sponge tissues. **A** For the sponge species analyzed across all locations the isotopic niche width of each species is indicated by the areas outlined by standard ellipse areas of overlap. Solid lines represent standard ellipse areas of overlap with same-colored dots representing individuals for each species. **B** Analysis of High Microbial Abundance (HMA) and Low Microbial Abundance (LMA) symbiotic states of Caribbean sponges. The isotopic niche width of each state is indicated by the areas outlined by standard ellipse areas of overlap with same-colored dots representing individuals for each symbiotic state.

The SIBER analysis, using both the δ^13^C and δ^15^N of the sponge tissues, shows the isotopic niche width for all sponges, grouped by individual species across all sampling sites and outlined by standard ellipses (Fig. 6a). Significant *p*-values generated from a residual permutation procedure, and Hotelling’s *T*^*2*^ test, using the corrected standard ellipse area, revealed that all pairwise comparisons between species were significant, with most species’ isotopic niche width overlaps ranging from 0–16% (Table S3). A significant 40% overlap in isotopic niche width for *A. compressa* and *A. tubulata/conifera* (Table S3, Fig. 6a) indicates a moderate degree of resource sharing between autotrophically derived DOM and POM, regardless of their symbiotic state (i.e., LMA versus HMA respectively).

When sponge tissue isotopic values are analyzed based on their HMA or LMA status (Fig. 6b), a significant effect of symbiotic state for δ^13^C (ANOVA: F_1,161_ = 70.26, *p* < 0.0001) was observed with HMA sponges having lower (−18.69 ± 0.083 [SE]) values than LMA sponges (−17.60 ± 0.097). No significant effects for δ^15^N (ANOVA: F_1,161_ = 13.78, *p* = 0.774) were detected between HMA (3.58 ± 0.117) and LMA (3.62 ± 0.090) sponges. Analysis of symbiotic state using SIBER showed a significant (Hotelling’s *T*^*2*^ = 70.77, F = 34.73, *p* = <0.001), but low, overlap of 14% in isotopic niche width between HMA and LMA sponges. Taking the isotopic results and the isotopic niche width analysis together suggests mixotrophy for both HMA and LMA sponges, and low levels of resource competition for DOM and POM between HMA and LMA symbiotic states.

## Discussion

### Microbiome composition and diversity

Early studies on the species richness and diversity of microbiomes for sponges from multiple habitats focused on enumerating differences between sponge species [19] and describing the presence of a core microbiota [19, 20]. Sponge microbiomes are often described as species-specific but do harbor many generalists as part of the core microbiota for a large range of hosts [9, 56]. Additionally, it appears that sponge microbiome communities are persistent and stable across the HMA-LMA dichotomy [57, 58]. Despite the presence of a core microbiota, key microbiome taxa can be used to identify the symbiotic state of sponges, and their functional attributes [9].

In this study, underlying host species differences in sponge microbiome diversity are significant, but are driven by sponge symbiotic state (i.e., HMA-LMA dichotomy), and not by geographic location as demonstrated here over large spatial scales across the Caribbean, and as previously reported for small spatial scales within a localized coral reef system [59]. In both studies the microbiome analysis, and comparison of HMA and LMA sponges, was not confounded by differences in environmental conditions. The HMA species used in this study contain *Thaumarchaeota*, *Cyanobacteria* (except *A. tubulata/conifera*)*, Poribacteria* and *Chloroflexi*, microbial taxa known to be functionally important for oxidizing ammonia, CO_2_-fixation by both oxygenic and non-oxygenic pathways, and complex carbohydrate degradation [60–62]. In sponges, both functional convergence and redundancy within their microbiomes has been observed [63, 64]. This facilitates the maintenance of metabolic pathways associated with carbon and nitrogen metabolism, chemical defense production, and complimentary pathways such as the biosynthesis of vitamins by symbionts and their catabolism by the host [63, 65, 66]. While Fan et al. [63] used both HMA and LMA sponge species in their study on the functionl redundancy and convergence in the metabolic pathways of sponge microbiomes, they did not contextualize their findings, as did Ribes et al. [64], within the HMA-LMA dichotomy.

The functional differences in overall metabolic capacity, assessed by quantifying the enrichment in KEGG pathways among the sponge metagenomes, was driven primarily by host species. However, sponge species differences in metabolic capacity, as was the case for differences in their microbiomes, are embedded within their symbiotic state with HMA species having greater overall functional metabolic capacity than LMA species [9]. It is interesting to note that the LMA sponge, *Niphates erecta* has a greater diversity of metagenomic pathways originating from the KEGG analysis of metazoan genes compared to all other sponges in this study. However, LMA species do show greater metabolic capacity than HMA species in some pathways such as carbohydrate metabolism and aerobic respiration, while HMA samples were enriched for metabolic processes involving nitrogen and sulfur cycling, as well as amino acid metabolism. In addition, the functional capacity of HMA sponges for the biosynthesis of secondary metabolites was highly enriched compared to LMA sponges. This is consistent with a recent analysis by Pankey et al. [9] that showed a significant increase in feeding deterrence among HMA sponges relative to LMA sponges over evolutionary timescales, indicating the functional importance of these biosynthetic pathways, and strong selection pressure to maintain them. In addition, some of these HMA species exhibit allelopathic and antimicrobial activity that would further their ecological resistance to competitors and/or pathogens in these systems [67, 68].

### Metagenomics and metabolic functional capacity of sponges

What are the differences in metabolic capacity between HMA and LMA sponges? As reported previously [9], the relative genomic contributions of the sponge host to the holobiont metabolic and biochemical capacity are greater in LMA compared to HMA sponges. In contrast, the relative contribution of the microbiome (*Archaea* and *Bacteria*) in HMA sponges to their holobiont functional capacity is consistently higher than for the LMA microbiome across most metabolic pathways. This is supported by the functional redundancy from diverse prokaryotes diagnostic of HMA sponges (i.e., *Actinobacteria, Chloroflexi, Nitrospirae, Spirochetes*). In addition, HMA sponges have significantly greater capacity for O-antigen, lipopolysaccharide and protein biosynthesis directly related to the maintenance, biosynthetic requirements and energetic costs associated with their higher densities of symbiotic prokaryotes. While prokaryotic carbon fixation (i.e., anaerobic autotrophic pathways) and oxygenic photosynthesis pathways (i.e., Calvin cycle) were marginally enriched in LMA symbiont communities, oxygenic photosynthesis enzymes accounted for a fraction of the reads compared to other carbon-fixing pathways for all sponges suggesting low autotrophic inputs from symbiont photosynthesis. A closer examination of the distribution of enzyme-mapped read counts among carbon-fixing pathway enzymes indicates that HMA and LMA sponges fix carbon via multiple mechanisms. HMA sponges were enriched for enzymes involved in the reductive citrate cycle, the 3-hydroxypropionate cycle and the hydroxyproprionate-hydroxybutyrate cycles (known primarily from *Chloroflexi* and aerobic *Archaea*), while LMA sponges were enriched for enzymes in the Wood-Ljungdahl cycle (characteristic of *Proteobacteria*) (Fig. S8). Given the diversity of photoautotrophic and chemoautotrophic carbon-fixing pathways in coral reef sponges, the autotrophic capacity of sponges should be further explored and their trophic status as potential mixotrophs quantified.

### Stable isotopes and sponge trophic ecology

The δ^13^C values of the sponges in this study range from −17.16 ± 0.12‰ (SE) to −18.80 ± 0.13‰ and were significantly different between HMA and LMA sponges. *Aplysina cauliformis* and *Xestospongia muta* exhibited the lowest δ^13^C values indicative of consuming more autotrophic (i.e., DOM; δ^13^C of DOM from either macrophytes or corals, −11.21 to −19.50‰ and −15.46 to −17.95‰, respectively [69]), compared to heterotrophic (i.e., POM; −17.78 to −27.67‰ [69]), sources. The δ^13^C values for these HMA sponges could come from either photosynthates released by cyanobacterial symbionts, or the consumption of DOM or POM from an autotrophic source. The stable isotope values gave no indication that CO_2_ fixation by alternate pathways, such as the reductive citrate acid cycle (−14.0‰), known to occur in the abundant *Chloroflexi* symbionts [62] changed the isotopic values of the sponges studied. For *X. muta* ^13^C-HCO^-3^ tracer studies have shown initial uptake by the microbiome, with subsequent translocation to, and equilibrium with, the host after a 12 h pulse-chase experiment [70], which has also been shown for *Chondrilla caribensis* using NanoSIMS, and where photoautotrophy was shown to contribute only 7% of the total daily carbon uptake [71]. Other studies using compound-specific isotopic analysis of amino acids (CSIA-AA) on sponges have shown little dependence on photoautotrophy for shallow and mesophotic sponges [72, 73], with either no dependence on POM [72], or the consumption of DOM and translocation of essential amino acids from the bacterial symbionts to the host, with or without POM consumption [72, 73].

The δ^15^N value is often used as a measure of heterotrophy or change in trophic position in animals, and in sponges it has been used to describe changes in trophic position with increasing depth [33]. The HMA and LMA sponge δ^15^N values were not significantly different in this study because sponges from each location were exposed to similar irradiances, temperatures and concentrations of food and nutrients [38]. The microbiomes of sponges have members involved in all pathways of a complete nitrogen cycle, both its aerobic and anaerobic components with its different fractionation factors [23, 66, 69]. This internal recycling could affect the δ^15^N values of sponges as nitrification and denitrification can increase δ^15^N values [74], while nitrogen fixation can decrease the δ^15^N values of sponges [23]. The δ^15^N values of sponges in this study are also consistent with the consumption of picoplankton including isotopically lighter nitrogen-fixing bacteria that would be available in shallow tropical waters [75]. Given that the likely isotopic half-life in sponges for these POM sources is ~2 mo [32], combined with the similar characteristics for each collection location, the observed consistency in δ^15^N values is not unexpected. In fact, if one takes a commonly used metabolic fractionation factor of 3.5‰ for δ^15^N from one trophic level to the next and subtracts that from the tissue δ^15^N values observed here, you find a range of δ^15^N values for sponges between −0.61‰ to 0.95‰ which suggests that the original food source for these sponges was influenced by nitrogen fixation [76]. This metric is not without its issues when applied to multi-compartmental mutualistic symbioses such as corals or sponges [76] but is informative as an initial estimate.

The trophic ecology of the sponges studied here was also quantified using a SIBER analysis of their δ^13^C and δ^15^N stable isotopic values [54]. More broadly, these species differences are embedded within a significant 14% isotopic niche overlap between HMA and LMA sponges across the Caribbean basin, revealing a very low degree of resource sharing between these symbiotic states [54] which consume mostly autotrophically sourced DOM or live POM, respectively [77]. In a similar study by Freeman et al. [78] a 31% isotopic niche overlap was observed between HMA and LMA sponges indicating greater resource sharing than reported here, and where there was no difference in isotopic niche space when all sponge species, including HMA species designated as low chlorophyll (HMA-L) or high chlorophyll (HMA-H) species, are analyzed together. Additionally, in the absence of any environmental data, either abiotic or biotic, the observations in Freeman et al. [78] for species, or HMA versus LMA differences, cannot be untangled from the possible confounding effects of different environments (e.g., trophic resources) at the sites within the Miskito Cays, Honduras. Here, *Agelas tubulata/conifera*, identified as an HMA sponge, sits in an isotopic niche space between HMA and LMA species where pairwise comparisons indicate significant differences between *A. tubulata/conifera* and all other sponge species, regardless of symbiotic state, as observed in previous studies [69, 79]. Additionally, the SIBER analysis places *A. tubulata/conifera* in a similar isotopic niche with LMA sponge *Amphimedon compressa*, despite the differences in their microbiomes. However, based on the metagenomic data, the functional capacity of *A. tubulata/conifera* is indistinguishable from other HMA sponges. One possible explanation is that *A. tubulata/conifera* has very few cyanobacterial reads, consistent with it being an HMA-L sponge [69] that depends more on POM, with its increased bioavailability [32]. While differences in host species have been invoked as driving the trophic biology of sponges [79], the same study identified a significant effect of symbiont state on the isotopic signature of a sponges with an *R*^*2*^ = 0.20 which is within the range between a medium and large effects size and therefore ecologically relevant.

### The microbiome of HMA and LMA sponges determines their trophic ecology

Do the diversity, abundance and metabolic capacity differences in the microbiomes of HMA and LMA sponges determine the trophic ecology of HMA and LMA holobionts [27, 36, 69, 80]? Both HMA and LMA sponges consume DOM and POM, but LMA sponges have higher pumping rates and greater consumption of POM [27, 36, 71]. Conversely, HMA sponges typically have lower pumping rates and greater consumption of DOM [27, 36, 76]. Notwithstanding concerns regarding the allometric effects of sponge size (i.e., volume, mass, or length) on pumping rates [81], which can be effectively addressed using statistical approaches [33], direct measurements on emergent sponges in the Caribbean show that HMA sponges acquired 71–93% of their carbon from DOM, whereas LMA sponges acquired only 0–5% of their carbon from DOM [77]. Additionally, *Agelas tubulata/conifera, and Xestospongia*, which were also part of this study, showed similar volumes and pumping rates in the McMurray et al. [77] study, suggesting that allometric effects did not significantly confound these results. Consistent with these differences between HMA and LMA sponges, Rix et al. [82] showed that DOM uptake by the microbiome of HMA sponges accounts for 65–87% of assimilated DOM and ~60% of the total heterotrophic inputs. Conversely, DOM assimilation by LMA microbiomes is <5% while the majority (>95%) of both DOM and POM assimilation is carried out by the host [82]. Importantly, the microbiomes of HMA sponges have been shown to reprocess DOM into free amino acids that are then translocated to the host [72, 73]. These free amino acids are readily available for protein synthesis and given the 2–3 orders more bacterial biomass in HMA sponges compared to LMA sponges this is a significant ecological advantage.

A recent analysis of sponge co-phylogeny between sponges and their microbiomes [9] showed that the microbial composition of HMA and LMA microbiomes across host species represent distinct ecological communities despite variations in the microbiomes of sponges that suggest intermediates in this dichotomy exist [79] or could represent evolutionary transitional states. Host microbiome differences were also specific for either HMA or LMA sponges with high endemism [9]. This is exemplified by the fact that sponges generally, and HMA holobionts specifically, exhibit a strong signal of phylosymbiosis and co-phylogeny with their microbiomes that have been shaped by natural selection to specialize during a period of rapid diversification on coral reefs in the Cenozoic [9, 83]. These specializations would include being able to exploit new, and abundant, trophic resources (i.e., DOM), and to produce secondary metabolites for defensive purposes [9]. The HMA sponges examined here, regardless of host taxonomy, also exhibit significantly greater metabolic functional capacity than their LMA counterparts that is attributable to their microbiomes. The data presented here provide strong support for sponge microbiomes driving their trophic ecology, and for the HMA-LMA dichotomy [9].

## Conclusions

How should we study the trophic ecology of sponges? The HMA-LMA dichotomy, based on multiple phenotypic characteristics described above, is principally understood in the context of a combined character state, microbiome community structure and biomass, which varies between HMA and LMA sponge species. The most common variant of this dichotomy is based on the presence of high or low concentrations of chl *a* from the presence of cyanobacteria in the microbiome [84] that is largely confined to HMA sponges [36]. While ecologically interesting, a recent study on the evolution of HMA and LMA sponges analyzed a very large data set of sponges and their microbiomes, including those considered HMA-L or HMA-H variants, and found that the presence of cyanobacteria was not diagnostic of HMA sponges in Random Forest models, with many LMA sponge communities characterized by high relative abundances of this bacterial phylum [9].

Another approach is to consider HMA and LMA sponges as two symbiotic states, or phenotypes. Simply, a phenotype is based on a set of observable/measurable characteristics of individuals resulting from the interaction of its genotype with the environment. Host sponge predisposition towards either HMA or LMA status likely represents an inherited trait (genotype) given the uniformity and consistently of communities within species and similarity in communities between closely related hosts. This genotype along with environmental variables determine the morphology and physiology of the host (ontogenetic phenotype), its microbiome (symbiotic phenotype), and ultimately holobiont function (multiple phenotypes). Both ontogenetic phenotypes, which are controlled by genetics of the developmental program of the host, and the response of the microbiome to the external and internal environment, can change because these phenotypes are plastic (i.e., phenotypic plasticity) as has been previously demonstrated for sponges [85, 86].

Ecologically, sponges represent a dominant functional group on coral reefs worldwide, and there is evidence that sponge abundance and biomass are increasing on shallow reefs as coral cover declines due to anthropogenic disturbances [5]. All sponges consume varying amounts of DOM and POM, and the phenotypic differences described here for select sponge species across a wide geographic expanse in the Caribbean basin have important ecological ramifications for the distribution of HMA and LMA sponges. This includes sufficient trophic niche separation within complex sponge communities to increase local sponge biodiversity and co-existence [87]. Increased DOM production caused by phase shifts to algal-dominated coral reefs has the potential to facilitate selection for increasing sponge populations generally, and chemically defended HMA sponges that can take advantage of the increasing amounts of DOM on coral reefs [9]. In the Anthropocene, the potential for this ecological shift will have to be reconciled with potential decreases in both DOM and POM due to predicted reductions of phytoplankton and picoplankton productivity in the future [8]. This is especially relevant to the sponge loop and the flux of POM in the form of detritus onto coral reefs [28, 29], because the consumption of both DOM and POM contribute to detritus production by sponges on tropical coral reefs [88].

## Supplementary information

Supplemental Material

## Data Availability

Microbial 16 S rRNA MiniSeq reads and metagenomic libraries are available at the NCBI Short Read Archive under BioProject PRJNA555077. Complete bioinformatic pipeline including scripts is available through the GitHub repository https://github.com/scriptomika/SpongeDOB.
