# Supplementary material for: Microbiome diversity and metabolic capacity determines the trophic ecology of the holobiont in Caribbean sponges
Source: ISME Commun. 2022 Nov 10;2:112. doi: 10.1038/s43705-022-00196-3 (PMC9723761; doi:10.1038/s43705-022-00196-3)
Supplement: Supplementary file 1 — Supplemental Material [file 43705_2022_196_MOESM1_ESM.docx]

**Supplemental Information**

**Microbiome Diversity and Metabolic Capacity Determines the Trophic Ecology of the Holobiont in Caribbean Sponges**

Michael P. Lesser^1*^, M. Sabrina Pankey^1^, Marc Slattery^2^, Keir J Macartney^1,3^, Deborah J. Gochfeld^4^

^1^Department of Molecular, Cellular and Biomedical Sciences, University of New Hampshire, Durham, NH 03824 USA

^2^Department of BioMolecular Sciences, Division of Pharmacognosy, University of Mississippi, Oxford, MS 38677 USA

^3^Present address: University of Texas Rio Grande Valley, School of Earth, Environmental and Marine Sciences, Port Isabel, TX 78958 USA

^4^National Center for Natural Products Research, University of Mississippi, Oxford, MS 38677 USA

^*^Corresponding author: Michael P. Lesser; email: mpl@unh.edu


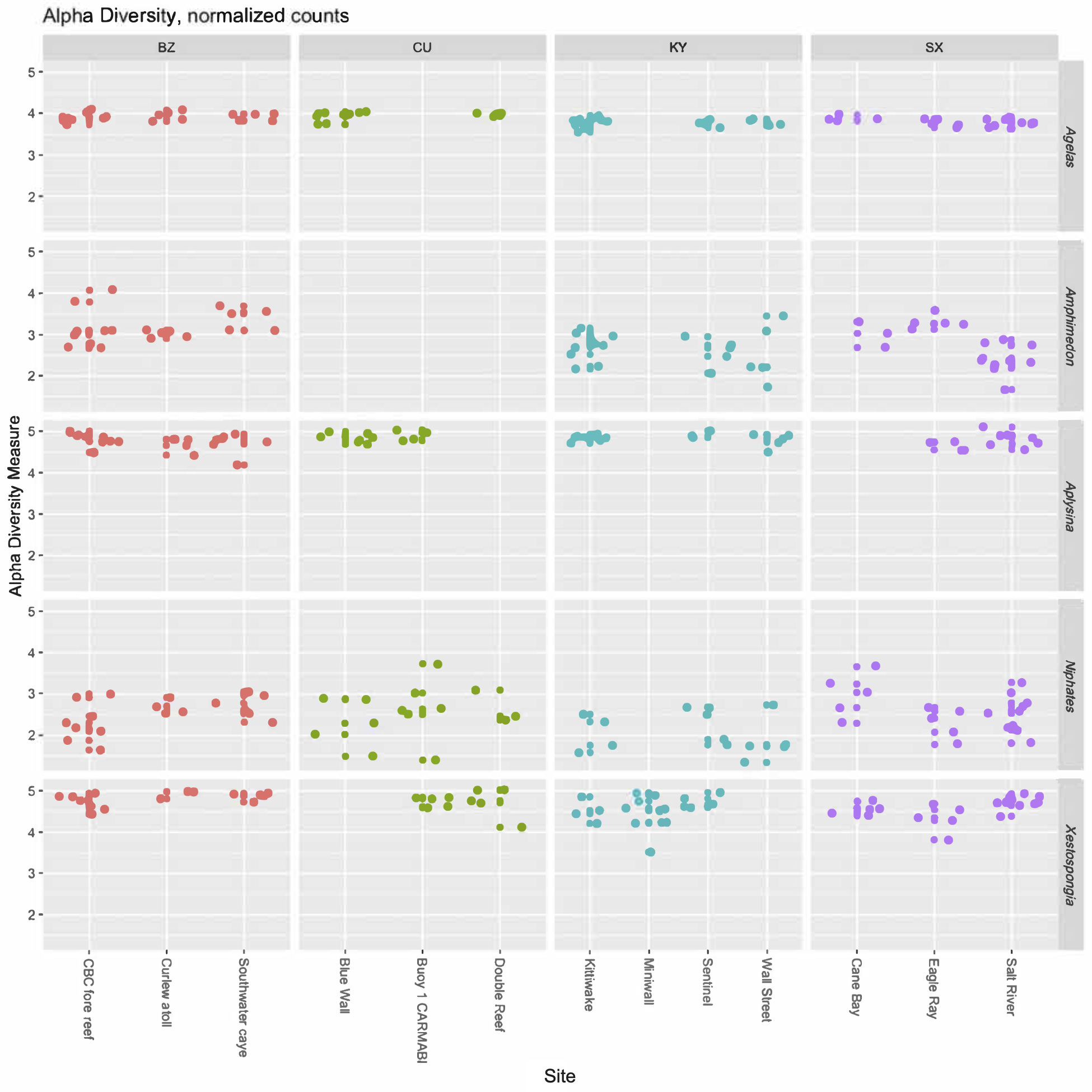


Figure S1. Shannon diversity values of alpha diversity in sponge microbial communities from different genera and reef sites. Location codes along the top (BZ=Belize, CU= Curaçao, KY=Grand Cayman, SX=St. Croix). HMA sponges, Agelas, Aplysina and Xestopongia. LMA sponges, Amphimedon and Niphates.


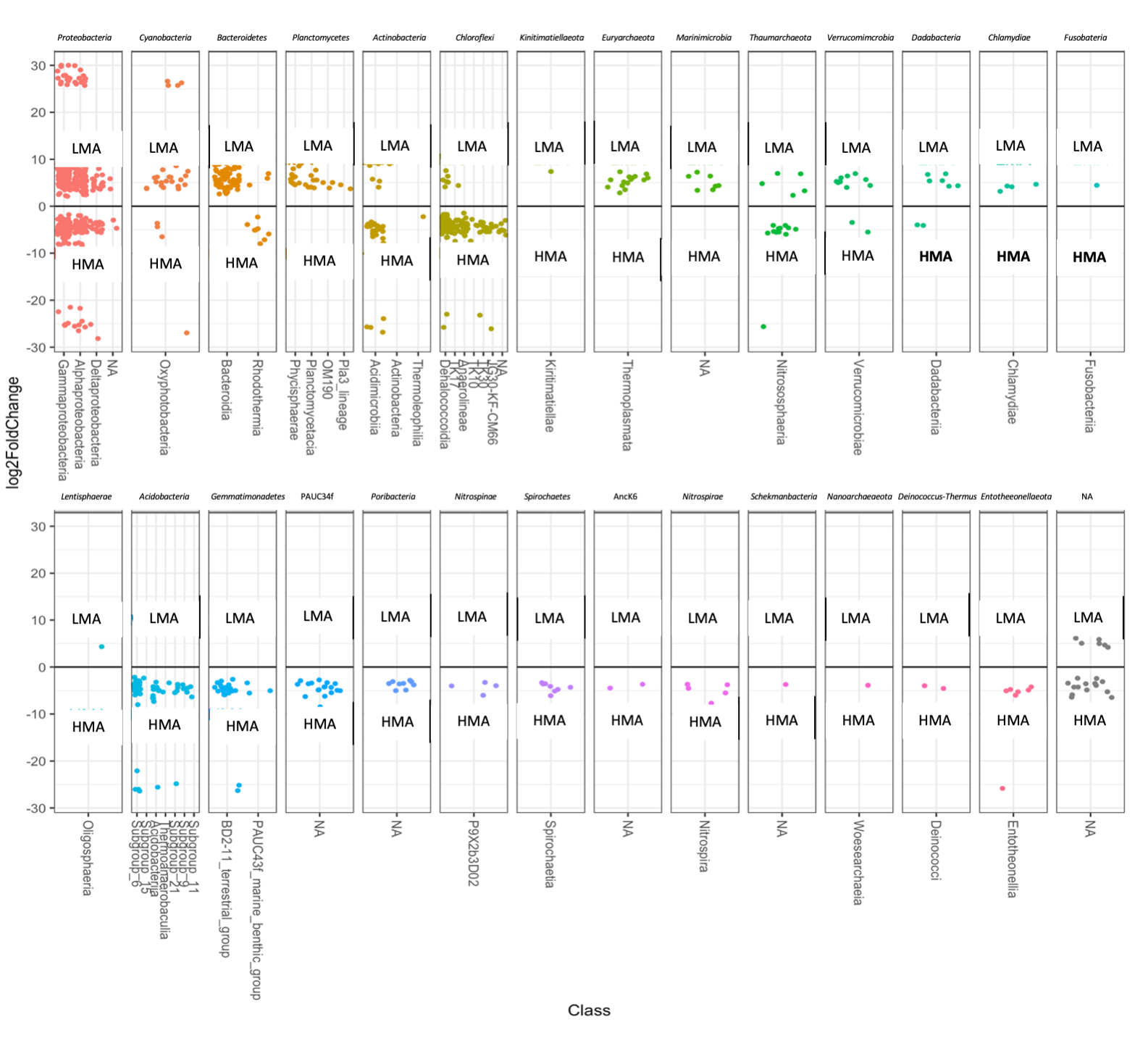


Figure S2. ASV enrichment in HMA versus LMA sponges. Significantly HMA-enriched are logFC<0; LMA-enriched logFC>0. Points represent ASV organized by microbial class, then grouped by phylum.

Figure S3. Shannon diversity of KEGG enzyme-mapped read counts originating from metazoan, archaeal and bacterial reads across sponge species, with symbiotic state noted, and location (BZ=Belize, CU= Curaçao, KY=Grand Cayman, SX=St. Croix).

Figure S4. Comparison of average relative taxonomic contributions of metagenomic reads to KEGG pathways in HMA and LMA samples. Metagenomic reads were assigned to NCBI taxonomic ranks and KEGG metabolic pathways using PALADIN. Circle size represents each domains relative contribution of reads to each major metabolic pathway category. Those cases where the red circles are larger than the blue ones indicate taxa which contribute more reads to the enzymes in that pathway for HMA (red) and LMA (blue) sponges.

Figure S5. Mean percentage representation of the enzyme-mapped read counts (left) and their relative abundances (right) for KEGG based metabolic pathways in HMA (red) and LMA (blue) sponge samples.

Figure S6. Differential enrichment of enzyme-mapped read counts participating in the four major prokaryotic carbon-fixation cycles. Mean fold-change (log2) indicated by color scale, where enzymes with higher proportions of mapped reads in red for HMA or blue for LMA sponges.

Figure S7. Abundances of genomic clusters encoding secondary metabolite synthesis pathways detected by AntiSMASH in each sponge sample. HMA samples shown in red; LMA in blue. Circle size indicates read abundance relative to overall sample reads mapping to metagenome assembly.


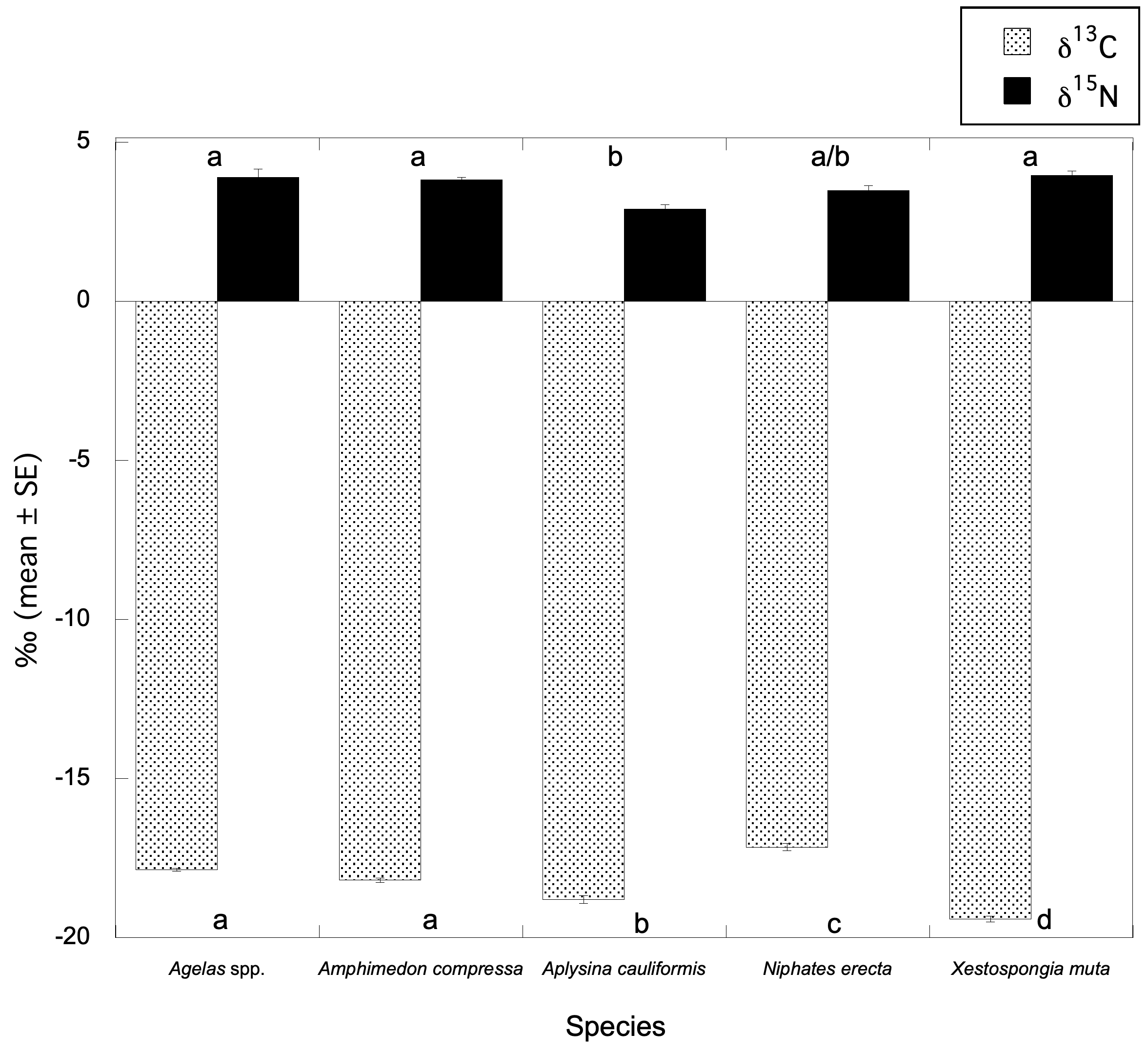


Figure S8. δ^13^C analysis and δ^15^N analysis of sponge species across all locations. Species with common superscripts are not significantly different from each other using Tukey’s HSD *post hoc* multiple comparison tests.

Table S1. Sponge sample collection location, reef site and GPS coordinates.

| ***Location*** | ***Reef Site*** | ***GPS Coordinates*** |
| --- | --- | --- |
| Grand Cayman | Kittiwake Anchor Chain | 19° 21.718'N, 81° 24.138'W |
| Grand Cayman | Sentinel Rock | 19° 22.075'N, 81° 24.990'W |
| Grand Cayman | Slaughterhouse Wall | 19° 21.776'N, 81° 24.250'W |
| Grand Cayman | Hepp's Pipeline | 19° 23.071'N, 81° 24.940'W |
| Curaçao | Blue Wall | 12° 8.1383'N, 68° 59.5583'W |
| Curaçao | Buoy 1 | 12° 07.33′N, 69° 03.8′W |
| Curaçao | Double Reef | 12° 06.402'N, 68° 56.932'W |
| Curaçao | Pescadero Bay | 12° 07.337'N,68° 58.285'W |
| St. Croix | Cane Bay | 17° 46.433'N, 64° 48.810'W |
| St. Croix | Eagle Ray | 17° 45.687'N, 64° 41.934'W |
| St. Croix | Salt River | 17° 47.069'N, 64° 46.020'W |
| Belize | Carrie Bow Cay | 16° 48.005'N, 88° 04.668'W |
| Belize | Curlew Cay | 16° 47.350'N, 88° 04.571'W |
| Belize | Southwater Cay | 16° 48.986'N, 88° 04.629'W |

| Table S2. KEGG pathways detected in metagenome analysis. Total enzymes detected and the total number of reads mapped across samples are provided. | | | | | | | | |  | | | |  |
| --- | --- | --- | --- | --- | --- | --- | --- | --- | --- | --- | --- | --- | --- |
| FDR q-values are provided for significant (q<0.05) pathways tested with PERMANOVA for effect of site, species, and symbiotic state (HMA/LMA) | | | | | | | | | | |  |  |  |
| Results of DESeq2 differential enrichment tests shown for significant pathways only: log2FC>0 indicates LMA-enriched (blue), log2FC<0 HMA-enriched (red). | | | | | | |  | | | | | |  |
| Metabolic process | Pathway | KEGG Pathway | Number enzymes | Reads mapped | q-values for PERMANOVA tests | | Differential Enrichment | | |  |  |  |  |
|  |  |  |  |  | species | symbiotic state | log2FoldChange (LMA/HMA) | Wald p-value (adj) | |  |  |  |  |
|  |  |  |  |  |  |  |  |  | |  |  |  |  |
| Amino acid metabolism | Selenocompound metabolism | ec00450 | 17 | 255230 | 0.001008065 | 0.001136364 | -0.804081154 | 1.91748E-35 | |  |  |  |  |
| Amino acid metabolism | Arginine biosynthesis | ec00220 | 31 | 372710 | 0.001008065 | 0.001136364 | -0.393210794 | 4.91743E-41 | |  |  |  |  |
| Amino acid metabolism | Phenylalanine metabolism | ec00360 | 61 | 227705 | 0.001008065 | 0.001136364 | -0.359381457 | 2.51401E-05 | |  |  |  |  |
| Amino acid metabolism | Cyanoamino acid metabolism | ec00460 | 29 | 78145 | 0.001008065 | 0.001136364 | -0.303612195 | 0.004459106 | |  |  |  |  |
| Amino acid metabolism | Cysteine and methionine metabolism | ec00270 | 85 | 829194 | 0.001008065 | 0.001136364 | -0.224702345 | 3.47144E-08 | |  |  |  |  |
| Amino acid metabolism | Phenylalanine tyrosine and tryptophan biosynthesis | ec00400 | 38 | 406235 | 0.001008065 | 0.001136364 | -0.152900065 | 0.000900206 | |  |  |  |  |
| Amino acid metabolism | Valine leucine and isoleucine biosynthesis | ec00290 | 14 | 501869 | 0.001008065 | 0.002173913 | 0.21269762 | 7.23304E-05 | |  |  |  |  |
| Amino acid metabolism | Lysine biosynthesis | ec00300 | 33 | 325432 | 0.001008065 | 0.001136364 | 0.274705286 | 2.9092E-07 | |  |  |  |  |
| Amino acid metabolism | Lysine degradation | ec00310 | 70 | 279546 | 0.001008065 | 0.001136364 | 0.30876796 | 8.72008E-10 | |  |  |  |  |
| Amino acid metabolism | beta-Alanine metabolism | ec00410 | 36 | 121684 | 0.001008065 | 0.001136364 | 0.368391389 | 4.48514E-06 | |  |  |  |  |
| Amino acid metabolism | Glycine serine and threonine metabolism | ec00260 | 69 | 812139 | 0.001008065 | 0.001136364 | 0.411410862 | 1.39904E-30 | |  |  |  |  |
| Amino acid metabolism | Taurine and hypotaurine metabolism | ec00430 | 20 | 108285 | 0.001008065 | 0.001136364 | 0.42854217 | 0.003009655 | |  |  |  |  |
| Amino acid metabolism | Valine leucine and isoleucine degradation | ec00280 | 36 | 396801 | 0.001008065 | 0.001136364 | 0.475954092 | 1.58643E-11 | |  |  |  |  |
| Amino acid metabolism | Tyrosine metabolism | ec00350 | 66 | 118687 | 0.001008065 | 0.001136364 | 0.829876373 | 2.15913E-13 | |  |  |  |  |
| Amino acid metabolism | Alanine aspartate and glutamate metabolism | ec00250 | 50 | 708864 | 0.001008065 | 0.001136364 |  |  | |  |  |  |  |
| Amino acid metabolism | Arginine and proline metabolism | ec00330 | 85 | 280002 | 0.001008065 | 0.001136364 |  |  | |  |  |  |  |
| Amino acid metabolism | Histidine metabolism | ec00340 | 39 | 298122 | 0.001008065 | 0.001136364 |  |  | |  |  |  |  |
| Amino acid metabolism | Tryptophan metabolism | ec00380 | 62 | 389708 | 0.001008065 | 0.001136364 |  |  | |  |  |  |  |
| Amino acid metabolism | Phosphote and phosphite metabolism | ec00440 | 30 | 16903 | 0.001008065 | 0.001136364 |  |  | |  |  |  |  |
| Amino acid metabolism | D-Amino acid metabolism | ec00470 | 51 | 123541 | 0.001008065 | 0.001136364 |  |  | |  |  |  |  |
| Amino acid metabolism | Glutathione metabolism | ec00480 | 39 | 422029 | 0.001008065 | 0.001136364 |  |  | |  |  |  |  |
| Biosynthesis secondary metabolites | Betalain biosynthesis | ec00965 | 3 | 326 |  |  | -5.423598934 | 4.55574E-13 | |  |  |  |  |
| Biosynthesis secondary metabolites | Biosynthesis of secondary metabolites - unclassified | ec00999 | 28 | 570 | 0.001008065 | 0.001136364 | -5.053451192 | 2.74312E-11 | |  |  |  |  |
| Biosynthesis secondary metabolites | Aflatoxin biosynthesis | ec00254 | 13 | 29444 |  |  | -4.908141115 | 2.87352E-29 | |  |  |  |  |
| Biosynthesis secondary metabolites | Flavone and flavonol biosynthesis | ec00944 | 32 | 271 |  |  | -4.587507396 | 7.32386E-05 | |  |  |  |  |
| Biosynthesis secondary metabolites | Isoquinoline alkaloid biosynthesis | ec00950 | 65 | 15237 | 0.001008065 | 0.001136364 | -4.153224967 | 5.25687E-39 | |  |  |  |  |
| Biosynthesis secondary metabolites | Flavonoid biosynthesis | ec00941 | 28 | 316 | 0.001008065 | 0.001136364 | -3.874456526 | 4.73174E-07 | |  |  |  |  |
| Biosynthesis secondary metabolites | Stilbenoid diarylheptanoid and gingerol biosynthesis | ec00945 | 12 | 86 |  |  | -3.649433517 | 0.001998654 | |  |  |  |  |
| Biosynthesis secondary metabolites | Neomycin kamycin and gentamicin biosynthesis | ec00524 | 28 | 4696 | 0.001008065 | 0.001136364 | -1.221059535 | 0.000290879 | |  |  |  |  |
| Biosynthesis secondary metabolites | Caffeine metabolism | ec00232 | 13 | 4070 | 0.001008065 | 0.001136364 | -0.99566694 | 0.010125393 | |  |  |  |  |
| Biosynthesis secondary metabolites | Acarbose and validamycin biosynthesis | ec00525 | 14 | 14926 | 0.001008065 | 0.001136364 | -0.971996705 | 0.003416114 | |  |  |  |  |
| Biosynthesis secondary metabolites | Phenylpropanoid biosynthesis | ec00940 | 32 | 48735 | 0.001008065 | 0.00529661 | -0.843307217 | 0.002484328 | |  |  |  |  |
| Biosynthesis secondary metabolites | Phezine biosynthesis | ec00405 | 13 | 41124 | 0.001008065 | 0.001136364 | -0.574805539 | 6.13671E-09 | |  |  |  |  |
| Biosynthesis secondary metabolites | Monobactam biosynthesis | ec00261 | 10 | 184862 | 0.001008065 | 0.001136364 | -0.098338392 | 0.008241682 | |  |  |  |  |
| Biosynthesis secondary metabolites | Novobiocin biosynthesis | ec00401 | 16 | 53196 | 0.001008065 | 0.001136364 | 0.440987502 | 0.025721048 | |  |  |  |  |
| Biosynthesis secondary metabolites | Penicillin and cephalosporin biosynthesis | ec00311 | 14 | 5454 | 0.001008065 | 0.001136364 | 1.596988054 | 7.83161E-07 | |  |  |  |  |
| Biosynthesis secondary metabolites | Clavulanic acid biosynthesis | ec00331 | 4 | 2093 |  |  | 2.730107715 | 0.000171837 | |  |  |  |  |
| Biosynthesis secondary metabolites | Staurosporine biosynthesis | ec00404 | 17 | 12405 | 0.001008065 | 0.001136364 | 6.615496895 | 1.03926E-22 | |  |  |  |  |
| Biosynthesis secondary metabolites | Carbapenem biosynthesis | ec00332 | 5 | 51713 |  |  |  |  | |  |  |  |  |
| Biosynthesis secondary metabolites | Streptomycin biosynthesis | ec00521 | 18 | 65033 | 0.001008065 | 0.001136364 |  |  | |  |  |  |  |
| Biosynthesis secondary metabolites | Isoflavonoid biosynthesis | ec00943 | 22 | 2 |  |  |  |  | |  |  |  |  |
| Biosynthesis secondary metabolites | Tropane piperidine and pyridine alkaloid biosynthesis | ec00960 | 27 | 51782 | 0.001008065 | 0.001136364 |  |  | |  |  |  |  |
| Biosynthesis secondary metabolites | Glucosinolate biosynthesis | ec00966 | 15 | 48468 |  |  |  |  | |  |  |  |  |
| Biosynthesis secondary metabolites | Biosynthesis of various secondary metabolites III | ec00997 | 32 | 64195 | 0.001008065 | 0.002173913 |  |  | |  |  |  |  |
| Biosynthesis secondary metabolites | Biosynthesis of various secondary metabolites II | ec00998 | 30 | 5434 | 0.001008065 | 0.001136364 |  |  | |  |  |  |  |
| Biosynthesis secondary metabolites | Acridone alkaloid biosynthesis | ec01058 | 3 | 30 |  |  |  |  | |  |  |  |  |
| Carbohydrate metabolism | Pentose and glucuronate interconversions | ec00040 | 73 | 127660 | 0.001008065 | 0.001136364 | -0.447777406 | 2.61353E-06 | |  |  |  |  |
| Carbohydrate metabolism | Glyoxylate and dicarboxylate metabolism | ec00630 | 76 | 613580 | 0.001008065 | 0.001136364 | -0.120204228 | 0.016608153 | |  |  |  |  |
| Carbohydrate metabolism | Citrate cycle TCA cycle) | ec00020 | 26 | 785637 | 0.001008065 | 0.001136364 | 0.154724739 | 9.85398E-06 | |  |  |  |  |
| Carbohydrate metabolism | Inositol phosphate metabolism | ec00562 | 48 | 114764 | 0.001008065 | 0.001136364 | 0.360929677 | 0.028647122 | |  |  |  |  |
| Carbohydrate metabolism | Pentose phosphate pathway | ec00030 | 55 | 372176 | 0.001008065 | 0.001136364 | 0.361848112 | 6.96936E-11 | |  |  |  |  |
| Carbohydrate metabolism | Butanoate metabolism | ec00650 | 61 | 377980 | 0.001008065 | 0.001136364 | 0.42482921 | 3.92649E-16 | |  |  |  |  |
| Carbohydrate metabolism | Ascorbate and aldarate metabolism | ec00053 | 57 | 65283 | 0.001008065 | 0.001136364 | 0.432641021 | 0.000145937 | |  |  |  |  |
| Carbohydrate metabolism | Galactose metabolism | ec00052 | 48 | 106555 | 0.001008065 | 0.001136364 | 0.438712319 | 7.52819E-09 | |  |  |  |  |
| Carbohydrate metabolism | Glycolysis / Gluconeogenesis | ec00010 | 50 | 674440 | 0.001008065 | 0.001136364 |  |  | |  |  |  |  |
| Carbohydrate metabolism | Fructose and mannose metabolism | ec00051 | 76 | 141729 | 0.001008065 | 0.001136364 |  |  | |  |  |  |  |
| Carbohydrate metabolism | Starch and sucrose metabolism | ec00500 | 76 | 141099 | 0.001008065 | 0.001136364 |  |  | |  |  |  |  |
| Carbohydrate metabolism | Amino sugar and nucleotide sugar metabolism | ec00520 | 127 | 352625 | 0.001008065 | 0.001136364 |  |  | |  |  |  |  |
| Carbohydrate metabolism | Pyruvate metabolism | ec00620 | 74 | 844267 | 0.001008065 | 0.001136364 |  |  | |  |  |  |  |
| Carbohydrate metabolism | Propanoate metabolism | ec00640 | 49 | 457167 | 0.001008065 | 0.001136364 |  |  | |  |  |  |  |
| Carbohydrate metabolism | C5-Branched dibasic acid metabolism | ec00660 | 23 | 231016 | 0.001008065 | 0.001136364 |  |  | |  |  |  |  |
| Cofactors and vitamins | Ubiquinone and other terpenoid-quinone biosynthesis | ec00130 | 45 | 86062 | 0.001008065 | 0.001136364 | -0.581147699 | 9.85398E-06 | |  |  |  |  |
| Cofactors and vitamins | Vitamin B6 metabolism | ec00750 | 29 | 114813 | 0.001008065 | 0.001136364 | -0.232898393 | 0.000764236 | |  |  |  |  |
| Cofactors and vitamins | Pantothete and CoA biosynthesis | ec00770 | 33 | 461423 | 0.001008065 | 0.001136364 | 0.109460333 | 0.007314647 | |  |  |  |  |
| Cofactors and vitamins | Lipoic acid metabolism | ec00785 | 5 | 48300 | 0.001008065 | 0.001136364 | 0.260683539 | 0.020693215 | |  |  |  |  |
| Cofactors and vitamins | Nicotite and nicotimide metabolism | ec00760 | 61 | 284876 | 0.001008065 | 0.001136364 | 0.381750107 | 2.5723E-11 | |  |  |  |  |
| Cofactors and vitamins | One carbon pool by folate | ec00670 | 27 | 397243 | 0.001008065 | 0.001136364 |  |  | |  |  |  |  |
| Cofactors and vitamins | Thiamine metabolism | ec00730 | 28 | 222932 | 0.001008065 | 0.001136364 |  |  | |  |  |  |  |
| Cofactors and vitamins | Riboflavin metabolism | ec00740 | 28 | 126243 | 0.001008065 | 0.002173913 |  |  | |  |  |  |  |
| Cofactors and vitamins | Biotin metabolism | ec00780 | 20 | 167561 | 0.001008065 | 0.001136364 |  |  | |  |  |  |  |
| Cofactors and vitamins | Folate biosynthesis | ec00790 | 52 | 219244 | 0.001008065 | 0.001136364 |  |  | |  |  |  |  |
| Cofactors and vitamins | Retinol metabolism | ec00830 | 13 | 8762 | 0.001008065 |  |  |  | |  |  |  |  |
| Cofactors and vitamins | Porphyrin and chlorophyll metabolism | ec00860 | 106 | 482727 | 0.001008065 | 0.001136364 |  |  | |  |  |  |  |
| Energy metabolism | Sulfur metabolism | ec00920 | 53 | 179132 | 0.001008065 | 0.001136364 | -0.35801519 | 6.3036E-06 | |  |  |  |  |
| Energy metabolism | Nitrogen metabolism | ec00910 | 39 | 224191 | 0.001008065 | 0.001136364 | -0.184108395 | 0.00548953 | |  |  |  |  |
| Energy metabolism | Carbon fixation in photosynthetic organisms | ec00710 | 25 | 383053 | 0.001008065 | 0.001136364 | 0.103845457 | 0.019778708 | |  |  |  |  |
| Energy metabolism | Carbon fixation pathways in prokaryotes | ec00720 | 51 | 899055 | 0.001008065 | 0.001136364 | 0.207756613 | 2.0028E-09 | |  |  |  |  |
| Energy metabolism | Methane metabolism | ec00680 | 91 | 563800 | 0.001008065 | 0.001136364 | 0.224600111 | 4.04393E-05 | |  |  |  |  |
| Energy metabolism | Photosynthesis | ec00195 | 3 | 142974 | 0.001008065 | 0.00529661 | 0.428517305 | 4.04393E-05 | |  |  |  |  |
| Energy metabolism | Oxidative phosphorylation | ec00190 | 10 | 485077 | 0.001008065 | 0.001136364 |  |  | |  |  |  |  |
| Glycan metabolism | O-Antigen nucleotide sugar biosynthesis | ec00541 | 63 | 134080 | 0.001008065 | 0.001136364 | -0.341959069 | 0.033761346 | |  |  |  |  |
| Glycan metabolism | Lipopolysaccharide biosynthesis | ec00540 | 30 | 157043 | 0.001008065 | 0.001136364 | -0.252413294 | 0.039231121 | |  |  |  |  |
| Glycan metabolism | Glycosphingolipid biosynthesis - globo and isoglobo series | ec00603 | 11 | 29855 | 0.001008065 |  | 0.506481935 | 0.045241083 | |  |  |  |  |
| Glycan metabolism | Glycosaminoglycan degradation | ec00531 | 15 | 36919 | 0.001008065 | 0.001136364 | 1.301654947 | 8.13512E-15 | |  |  |  |  |
| Glycan metabolism | Other glycan degradation | ec00511 | 9 | 44174 | 0.001008065 | 0.001136364 | 1.314382459 | 2.82318E-16 | |  |  |  |  |
| Glycan metabolism | Glycosphingolipid biosynthesis - ganglio series | ec00604 | 8 | 36221 |  |  | 1.379414516 | 2.20918E-13 | |  |  |  |  |
| Glycan metabolism | N-Glycan biosynthesis | ec00510 | 35 | 7512 | 0.001008065 | 0.002173913 |  |  | |  |  |  |  |
| Glycan metabolism | Mucin type O-glycan biosynthesis | ec00512 | 8 | 18 |  |  |  |  | |  |  |  |  |
| Glycan metabolism | Various types of N-glycan biosynthesis | ec00513 | 24 | 16767 | 0.006 |  |  |  | |  |  |  |  |
| Glycan metabolism | Other types of O-glycan biosynthesis | ec00514 | 14 | 6093 | 0.001008065 | 0.006302521 |  |  | |  |  |  |  |
| Glycan metabolism | Mannose type O-glycan biosynthesis | ec00515 | 9 | 5008 |  |  |  |  | |  |  |  |  |
| Glycan metabolism | Glycosaminoglycan biosynthesis - chondroitin sulfate / dermatan sulfate | ec00532 | 12 | 2119 |  |  |  |  | |  |  |  |  |
| Glycan metabolism | Glycosaminoglycan biosynthesis - keratan sulfate | ec00533 | 6 | 14 |  |  |  |  | |  |  |  |  |
| Glycan metabolism | Glycosaminoglycan biosynthesis - heparan sulfate / heparin | ec00534 | 11 | 2138 |  |  |  |  | |  |  |  |  |
| Glycan metabolism | Peptidoglycan biosynthesis | ec00550 | 23 | 395008 | 0.001008065 | 0.015625 |  |  | |  |  |  |  |
| Glycan metabolism | Glycosylphosphatidylinositol GPI)-anchor biosynthesis | ec00563 | 3 | 403 |  |  |  |  | |  |  |  |  |
| Glycan metabolism | Lipoarabinomann LAM) biosynthesis | ec00571 | 4 | 364 |  |  |  |  | |  |  |  |  |
| Glycan metabolism | Arabinogalactan biosynthesis - Mycobacterium | ec00572 | 7 | 29 |  |  |  |  | |  |  |  |  |
| Lipid metabolism | Ether lipid metabolism | ec00565 | 23 | 8440 | 0.001008065 | 0.001136364 | -4.294621359 | 1.73388E-05 | |  |  |  |  |
| Lipid metabolism | Steroid biosynthesis | ec00100 | 29 | 6379 | 0.001008065 | 0.001136364 | -3.933791012 | 6.17833E-12 | |  |  |  |  |
| Lipid metabolism | Arachidonic acid metabolism | ec00590 | 26 | 647 | 0.001008065 | 0.001136364 | -3.866485488 | 1.84345E-11 | |  |  |  |  |
| Lipid metabolism | Cutin suberine and wax biosynthesis | ec00073 | 9 | 6310 | 0.001008065 | 0.001136364 | -2.140487529 | 3.21476E-05 | |  |  |  |  |
| Lipid metabolism | alpha-Linolenic acid metabolism | ec00592 | 13 | 74659 | 0.001008065 | 0.001136364 | -0.468042978 | 7.77704E-09 | |  |  |  |  |
| Lipid metabolism | Fatty acid elongation | ec00062 | 13 | 98851 | 0.001008065 | 0.001136364 | -0.452052672 | 0.000121286 | |  |  |  |  |
| Lipid metabolism | Glycerophospholipid metabolism | ec00564 | 63 | 135338 | 0.001008065 | 0.001136364 | 0.363368662 | 0.000441186 | |  |  |  |  |
| Lipid metabolism | Fatty acid degradation | ec00071 | 31 | 189118 | 0.001008065 | 0.001136364 | 0.436969507 | 5.16525E-09 | |  |  |  |  |
| Lipid metabolism | Sphingolipid metabolism | ec00600 | 31 | 57977 | 0.001008065 | 0.001136364 | 0.965154863 | 8.23614E-07 | |  |  |  |  |
| Lipid metabolism | Primary bile acid biosynthesis | ec00120 | 18 | 3729 | 0.001008065 | 0.001136364 | 1.403064157 | 0.007294554 | |  |  |  |  |
| Lipid metabolism | Fatty acid biosynthesis | ec00061 | 17 | 257550 | 0.001008065 | 0.001136364 |  |  | |  |  |  |  |
| Lipid metabolism | Secondary bile acid biosynthesis | ec00121 | 17 | 1087 | 0.001008065 | 0.001136364 |  |  | |  |  |  |  |
| Lipid metabolism | Steroid hormone biosynthesis | ec00140 | 36 | 19572 | 0.001008065 | 0.003232759 |  |  | |  |  |  |  |
| Lipid metabolism | Glycerolipid metabolism | ec00561 | 45 | 148731 | 0.001008065 | 0.001136364 |  |  | |  |  |  |  |
| Lipid metabolism | Linoleic acid metabolism | ec00591 | 13 | 167 |  |  |  |  | |  |  |  |  |
| Lipid metabolism | Biosynthesis of unsaturated fatty acids | ec01040 | 14 | 26436 | 0.001008065 | 0.001136364 |  |  | |  |  |  |  |
| Nucleotide metabolism | Purine metabolism | ec00230 | 109 | 986017 | 0.001008065 | 0.001136364 |  |  | |  |  |  |  |
| Nucleotide metabolism | Pyrimidine metabolism | ec00240 | 64 | 629724 | 0.001008065 | 0.001136364 |  |  | |  |  |  |  |
| Terpenoids and polyketides | Sesquiterpenoid and triterpenoid biosynthesis | ec00909 | 79 | 7687 | 0.001008065 | 0.001136364 | -6.87604394 | 6.58464E-76 | |  |  |  |  |
| Terpenoids and polyketides | Biosynthesis of vancomycin group antibiotics | ec01055 | 10 | 6584 |  |  | -1.400455742 | 1.19881E-07 | |  |  |  |  |
| Terpenoids and polyketides | Geraniol degradation | ec00281 | 10 | 90574 | 0.001008065 | 0.001136364 | -0.398141569 | 0.000523401 | |  |  |  |  |
| Terpenoids and polyketides | Terpenoid backbone biosynthesis | ec00900 | 51 | 239846 | 0.001008065 | 0.001136364 | 0.383848599 | 2.19736E-09 | |  |  |  |  |
| Terpenoids and polyketides | Biosynthesis of ansamycins | ec01051 | 5 | 77853 |  |  | 1.197314532 | 4.63371E-14 | |  |  |  |  |
| Terpenoids and polyketides | Insect hormone biosynthesis | ec00981 | 9 | 12350 |  |  | 1.259514216 | 1.14724E-05 | |  |  |  |  |
| Terpenoids and polyketides | Biosynthesis of siderophore group nonribosomal peptides | ec01053 | 12 | 11480 | 0.001008065 |  | 1.428408227 | 0.004830964 | |  |  |  |  |
| Terpenoids and polyketides | Tetracycline biosynthesis | ec00253 | 9 | 13 |  |  |  |  | |  |  |  |  |
| Terpenoids and polyketides | Biosynthesis of 12- 14- and 16-membered macrolides | ec00522 | 19 | 348 |  |  |  |  | |  |  |  |  |
| Terpenoids and polyketides | Polyketide sugar unit biosynthesis | ec00523 | 27 | 28750 | 0.001008065 | 0.001136364 |  |  | |  |  |  |  |
| Terpenoids and polyketides | Monoterpenoid biosynthesis | ec00902 | 49 | 36 |  |  |  |  | |  |  |  |  |
| Terpenoids and polyketides | Limonene and pinene degradation | ec00903 | 16 | 64736 | 0.001008065 | 0.001136364 |  |  | |  |  |  |  |
| Terpenoids and polyketides | Brassinosteroid biosynthesis | ec00905 | 2 | 242 |  |  |  |  | |  |  |  |  |
| Terpenoids and polyketides | Carotenoid biosynthesis | ec00906 | 44 | 10418 | 0.001008065 | 0.001136364 |  |  | |  |  |  |  |
| Terpenoids and polyketides | Zeatin biosynthesis | ec00908 | 10 | 19883 |  |  |  |  | |  |  |  |  |
| Terpenoids and polyketides | Biosynthesis of type II polyketide products | ec01057 | 27 | 52 |  |  |  |  | |  |  |  |  |
| Translation | Aminoacyl-tR biosynthesis | ec00970 | 31 | 1092664 | 0.001008065 | 0.001136364 |  |  | |  |  |  |  |
| Xenobiotics metabolism | Furfural degradation | ec00365 | 3 | 257 | 0.001008065 | 0.001136364 | -4.429845681 | 3.30505E-06 | |  |  |  |  |
| Xenobiotics metabolism | Atrazine degradation | ec00791 | 11 | 34156 | 0.001008065 | 0.001136364 | -2.891227646 | 8.03587E-30 | |  |  |  |  |
| Xenobiotics metabolism | Xylene degradation | ec00622 | 22 | 8741 | 0.001008065 | 0.001136364 | -2.271609441 | 2.25183E-12 | |  |  |  |  |
| Xenobiotics metabolism | Styrene degradation | ec00643 | 19 | 66503 | 0.001008065 | 0.001136364 | -0.852492349 | 9.89127E-06 | |  |  |  |  |
| Xenobiotics metabolism | Fluorobenzoate degradation | ec00364 | 14 | 15115 | 0.001008065 | 0.001136364 | -0.75695436 | 0.003641313 | |  |  |  |  |
| Xenobiotics metabolism | Aminobenzoate degradation | ec00627 | 51 | 126172 | 0.001008065 | 0.001136364 | -0.605996883 | 1.99577E-14 | |  |  |  |  |
| Xenobiotics metabolism | Chlorocyclohexane and chlorobenzene degradation | ec00361 | 26 | 24367 | 0.001008065 | 0.001136364 | -0.524047478 | 0.005100854 | |  |  |  |  |
| Xenobiotics metabolism | Caprolactam degradation | ec00930 | 22 | 81847 | 0.001008065 | 0.001136364 | -0.398861823 | 0.00117774 | |  |  |  |  |
| Xenobiotics metabolism | Benzoate degradation | ec00362 | 70 | 188268 | 0.001008065 | 0.001136364 | 0.234119046 | 0.000214374 | |  |  |  |  |
| Xenobiotics metabolism | Drug metabolism - other enzymes | ec00983 | 25 | 363811 | 0.001008065 | 0.002173913 | 0.277522642 | 2.62606E-05 | |  |  |  |  |
| Xenobiotics metabolism | Nitrotoluene degradation | ec00633 | 7 | 12891 | 0.001008065 | 0.001136364 | 1.204853759 | 1.2104E-05 | |  |  |  |  |
| Xenobiotics metabolism | Metabolism of xenobiotics by cytochrome P450 | ec00980 | 10 | 28391 | 0.001008065 | 0.001136364 | 1.468865435 | 1.2173E-10 | |  |  |  |  |
| Xenobiotics metabolism | Drug metabolism - cytochrome P450 | ec00982 | 8 | 28765 | 0.001008065 | 0.001136364 | 1.573464895 | 1.8369E-12 | |  |  |  |  |
| Xenobiotics metabolism | Bisphenol degradation | ec00363 | 3 | 4106 |  |  | 3.048018536 | 2.12678E-15 | |  |  |  |  |
| Xenobiotics metabolism | Dioxin degradation | ec00621 | 13 | 10666 | 0.001008065 | 0.001136364 |  |  | |  |  |  |  |
| Xenobiotics metabolism | Toluene degradation | ec00623 | 27 | 30311 | 0.001008065 | 0.001136364 |  |  | |  |  |  |  |
| Xenobiotics metabolism | Polycyclic aromatic hydrocarbon degradation | ec00624 | 21 | 6872 | 0.001008065 | 0.001136364 |  |  | |  |  |  |  |
| Xenobiotics metabolism | Chloroalkane and chloroalkene degradation | ec00625 | 17 | 35739 | 0.001008065 | 0.001136364 |  |  | |  |  |  |  |
| Xenobiotics metabolism | phthalene degradation | ec00626 | 9 | 28225 | 0.001008065 | 0.001136364 |  |  | |  |  |  |  |
| Xenobiotics metabolism | Ethylbenzene degradation | ec00642 | 6 | 31023 | 0.001008065 | 0.001136364 |  |  | |  |  |  |  |
| Xenobiotics metabolism | Steroid degradation | ec00984 | 13 | 358 | 0.001008065 | 0.024793388 |  |  | |  |  |  |  |

Table S3. SIBER analysis, using the δ^13^C and δ^15^N of sponge tissues, evaluates the isotopic niche width for all sponges identified by symbiotic phenotype and grouped by species, across all sampling locations (see Fig. 6 a). Proportion of overlap calculated using SEAc metrics from SIBER analysis for each species.

|  | *Agelas spp* | *Amphimedon comperessa* | *Aplysina cauliformis* | *Niphates erecta* | *Xestospongia muta* |
| --- | --- | --- | --- | --- | --- |
| *Agelas spp* (HMA) |  | 0.14 | 0.009 | 0.16 | 0 |
| *Amphimedon comperessa* (LMA) | 0.4 |  | 0.01 | 0 | 0 |
| *Aplysina cauliformis* (HMA) | 0.006 | 0.006 |  | 0 | 0.001 |
| *Niphates erecta* (LMA) | 0.11 | 0 | 0 |  | 0 |
| *Xestospongia muta* (HMA) | 0 | 0 | 0.005 | 0 |  |

Pairwise species comparisons for SIBER analysis of isotopic niche space overlap.

| **Species** | **Hotelling’s *T^2^*** | | **F-value** | | ***p*-value** | |
| --- | --- | --- | --- | --- | --- | --- |
| *Agelas-Amphimedon* | 15.62 | 7.422 | | <0.001 | |  |
| *Agelas-Aplysina* | 57.13 | 27.26 | | <0.001 | |  |
| *Agelas-Niphates* | 30.98 | 14.81 | | <0.001 | |  |
| *Agelas-Xestospongia* | 223.16 | 106.51 | | <0.001 | |  |
| *Aplysina-Niphates* | 115.66 | 55.31 | | <0.001 | |  |
| *Aplysina-Amphimedon* | 41.14 | 19.54 | | <0.001 | |  |
| *Aplysina-Xestospongia* | 51.06 | 24.37 | | <0.001 | |  |
| *Niphates-Amphimedon* | 49.94 | 23.79 | | <0.001 | |  |
| *Niphates-Xestospongia* | 226.81 | 108.47 | | <0.001 | |  |
| *Amphimedon-Xestospongia* | 106.11 | 50.4 | | <0.001 | |  |
